# Enhancing visual perception by modulating prestimulus alpha and beta power with tRNS

**DOI:** 10.1038/s42003-025-08600-z

**Published:** 2025-08-08

**Authors:** Jinwen Wei, Huiru Zou, Qianyuan Tang, Ziqing Yao, Gan Huang, Zhen Liang, Li Zhang, Lijie Ren, Xiaodong Cai, Chen Yao, Changsong Zhou, Zhiguo Zhang

**Affiliations:** 1 School of Intelligence Science and Engineering, Harbin Institute of Technology, Shenzhen, Guangdong China; 2 School of Biomedical Engineering, Medical School, Shenzhen University, Shenzhen, China; 3 Department of Physics, Centre for Nonlinear Studies and Beijing-Hong Kong-Singapore Joint Centre for Nonlinear and Complex Systems (Hong Kong), Institute of Computational and Theoretical Studies, and Life Science Imaging Centre, Hong Kong Baptist University, Kowloon Tong, Hong Kong, China; 4 Department of Psychology, The University of Hong Kong, Hong Kong, China; 5 Department of Neurology, Department of Neurosurgery, The National Key Clinic Specialty, Shenzhen Key Laboratory of Neurosurgery, the First Affiliated Hospital of Shenzhen University, Shenzhen Second People’s Hospital, and Shenzhen Clinical Research Center for Neurological Diseases, Shenzhen, China; 6 Peng Cheng Laboratory, Shenzhen, China

**Keywords:** Perception, Visual system

## Abstract

Visual variability is linked to prestimulus alpha (8–13 Hz) and beta (13–30 Hz) power fluctuations, yet their causal role remains unclear. Using transcranial random noise stimulation (tRNS), we tested whether externally modulating cortical excitability could influence these oscillations and alter perception. In a sham-controlled, within-subject design, 29 participants completed a visual detection task combined with electroencephalography (EEG) and functional near-infrared spectroscopy (fNIRS) recordings. Mental fatigue was modelled as a state-dependent factor. tRNS, primarily under low fatigue, increased online oxyhemoglobin (HbO) amplitude, suppressed offline prestimulus alpha and beta power, and reduced offline visual contrast threshold (VCT), indicating enhanced perception. Further analyses revealed that fatigue influenced the oscillations’ responsiveness to tRNS, and that under low fatigue, alpha power, more than beta, demonstrated greater functional sensitivity to VCT. These findings demonstrate that tRNS can improve perception by modulating alpha/beta oscillations in specific brain states, highlighting the role of brain state in neuromodulation efficacy.

## Abstract (web summary)

tRNS modulates prestimulus alpha and beta power to enhance visual perception in a state-dependent manner, revealing a causal role of low-frequency oscillations under low fatigue in shaping sensory processing.

## Introduction

Variability in visual perception in response to consistent stimuli remains a significant topic of interest in neuroscience. This phenomenon is exemplified by an observer’s fluctuating ability to detect near-threshold visual contrast stimuli across different trials^1^. Previous research has demonstrated that these perceptual inconsistencies are linked to prestimulus (or spontaneous) low-frequency neural oscillations. While foundational studies have historically focused more on alpha, both alpha (8–13 Hz) and beta (13–30 Hz) rhythms are thought to influence perceptual sensitivity^2–12^.

However, despite the temporal precedence of these neural oscillations in visual perception, current studies have primarily provided correlational rather than causal evidence for their link^1,8^. In other words, it remains unknown whether alpha and beta power in the prestimulus interval causally affect visual perception. This gap in knowledge is due to the predominant use of non-invasive neural imaging techniques, such as electroencephalography (EEG) and functional magnetic resonance imaging (fMRI), which primarily establish correlational relationships between neural features and behaviors^13^. The neuroscience community has emphasized the necessity of establishing causal relationships between physiological features (e.g., neural oscillations) and cognitive behaviors (e.g., visual perception)^14,15^. Establishing causality would significantly enhance our understanding of neural mechanisms and has important implications for rehabilitation and therapeutic interventions^16^.

Transcranial electrical stimulation (tES), a non-invasive neuromodulation technique, has significantly advanced our exploration of causal relationships between neural dynamics and cognitive functions^17^. Transcranial random noise stimulation (tRNS), in particular, has been shown to enhance cortical excitability^18^, and improve visual perception^19^. By boosting cortical excitability—which has been shown to relate closely to EEG alpha^11,20^ and beta^21,22^ power—tRNS offers a promising means to test whether changes in these oscillations play a causal role in visual perception.

However, two challenges remain when using tRNS in practical applications. First, recent research has shown that the efficacy of tES is influenced by specific neural states, such as arousal^23^, sleepiness^24^, and ongoing oscillations^25^. This state-dependent effect of tES is believed to contribute to the variability in modulation outcomes across studies^26^. Therefore, it is crucial to consider the influence of brain states during tRNS application when establishing a causal link between prestimulus alpha/beta power and visual perception. Second, studying the online effects of tES on neural oscillations is challenging due to the contamination of EEG recordings by tES artifacts^27^. Hemodynamic responses measured by fMRI or functional near-infrared spectroscopy (fNIRS) are less susceptible to these artifacts^13^. Hemodynamic changes are closely linked to neural activity through neurovascular coupling, where increases in cortical activity correlate with enhanced hemodynamic responses^28^. Therefore, we incorporated fNIRS to assess cortical excitability during tRNS, which temporally precedes and may contribute to the suppression of post-stimulation alpha/beta oscillations observed in EEG. This design enables a comprehensive examination of both online and offline effects of tRNS.

We hypothesized that tRNS would modulate both online hemodynamic responses measured by fNIRS and offline prestimulus alpha and beta power measured by EEG, thereby influencing visual perception in a state-dependent manner. Specifically, we aimed to establish a causal relationship between prestimulus alpha/beta oscillations and visual perception by demonstrating that tRNS-induced changes in these oscillations lead to altered perceptual performance, depending on the mental fatigue state.

**Fig. 1:**
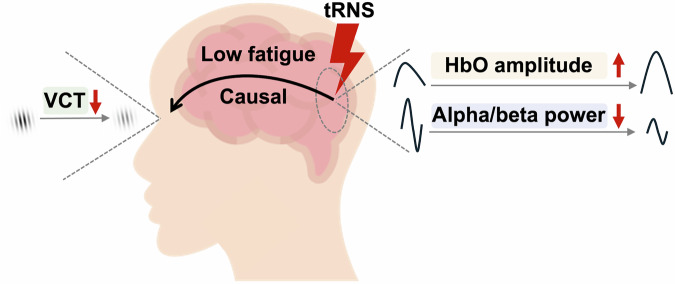
A schematic summary of our key findings. Under low fatigue, occipital tRNS increased online fNIRS HbO amplitude, decreased subsequent prestimulus EEG alpha and beta power, and reduced the VCT, providing causal evidence between prestimulus alpha/beta power and visual perception.

In this study, we utilized a tRNS-based experimental framework, integrating fNIRS and EEG with a visual detection task in a sham-controlled, single-blind, within-subject design. Consistent with our prior findings^29^, we categorized data into low and high fatigue states to examine how mental state modulates tRNS effects. Our findings show that occipital tRNS, primarily under low-fatigue conditions, increased online fNIRS oxyhemoglobin (HbO) amplitude, decreased subsequent prestimulus EEG alpha and beta power, and reduced the visual contrast threshold (VCT), suggesting a causal link between these oscillations and visual perception (see a schematic summary in Fig. 1). Further analyses indicate that fatigue shapes the brain’s responsiveness to stimulation and alters the functional sensitivity of alpha and beta activity to visual contrast detection.

## Results

### tRNS Affects HbO amplitude without considering fatigue

**Fig. 2:**
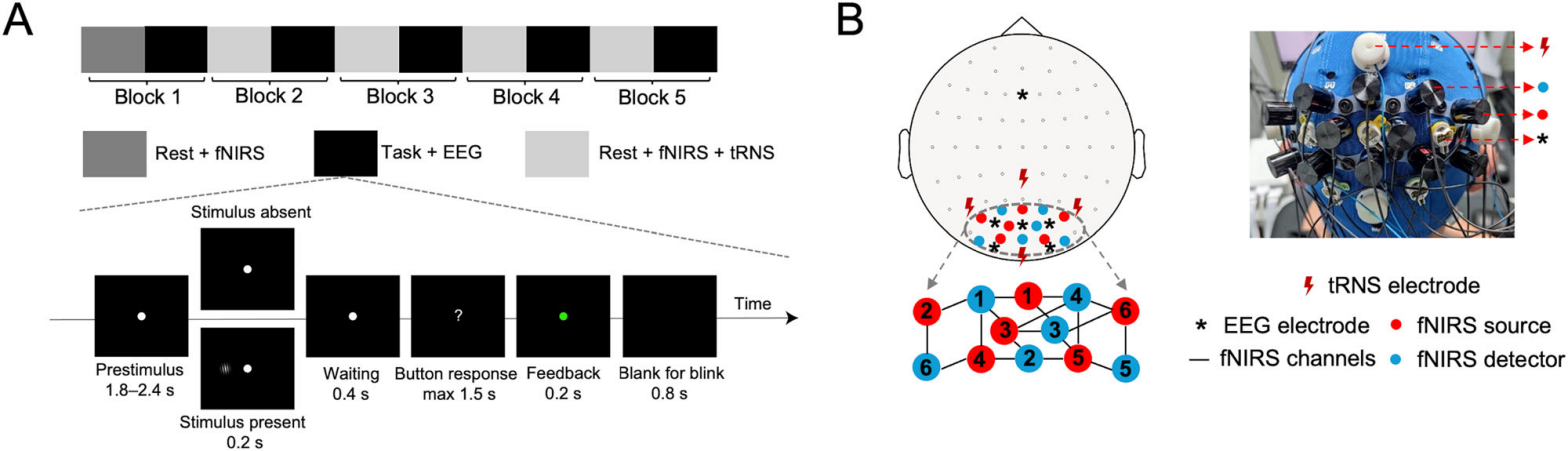
Experimental setup. **A** Each of the five blocks consisted of a rest period followed by a visual detection task. fNIRS was recorded during all rest sessions, and tRNS was applied during rest in Blocks 2–5 but not in Block 1, which served as a baseline. EEG was recorded during the task in all blocks, each compromising 80 trials. In 60% of trials, participants were presented with a near-threshold visual stimulus (a Gabor patch) for 0.2 s on either the left or right side of a central fixation dot (50% probability for each side). After a 0.4 s interval, the fixation dot changed to a question mark, prompting participants to indicate whether they perceived the stimulus by pressing a button. Feedback was provided via a green (correct) or red (incorrect) dot for 0.2 s, followed by a blank screen for blinking. The next trial began after a variable interval of 1.8–2.4 s. **B** EEG signals were recorded from occipital electrodes: O1, O2, PO3, POz, and PO4, with FCz as the reference electrode. Stimulation electrodes were placed over CPz, P5, P6, and Oz. Two stimulation conditions—sham and tRNS—were implemented in a counterbalanced, single-blind, within-subject design on two different days. fNIRS sources and detectors were positioned between tRNS and EEG electrodes (see Methods for details).

To evaluate the potential causal role of prestimulus alpha and beta power in visual perception, we conducted a visual detection task combined with fNIRS/EEG recording and tRNS in 29 healthy participants (15 females; mean age, 22.7 ± 1.9 years). Participants performed a visual detection task where Gabor patches with near-threshold contrasts were presented with a probability of 60% (Fig. 2A). The experiment consisted of five blocks, each separated by a 5 min rest interval. Each block comprised 80 trials, resulting in approximately 48 trials with stimulus presentation per block (80 × 60%). Visual contrast threshold (VCT) was estimated within each block using a cumulative Gaussian function following our previous study^29^. Each participant completed the task twice—once with tRNS and once with sham stimulation—with an interval of 3–7 days between sessions.

Fatigue ratings were collected before and after each block using a 7-point scale, and the average of the two ratings was used to index the prevailing fatigue state for that block. EEG data were recorded during the visual task in every block, while fNIRS data were collected over the occipital lobe during the preceding rest periods. tRNS, delivered at a high-frequency band of 100–640 Hz, was applied concurrently with fNIRS during rest in Blocks 2–5 (Fig. 2A, B). Notably, fNIRS data were recorded during the actual application of tRNS, whereas EEG data were collected after stimulation, ensuring no overlap between stimulation and EEG recording (Fig. 2A).

**Fig. 3:**
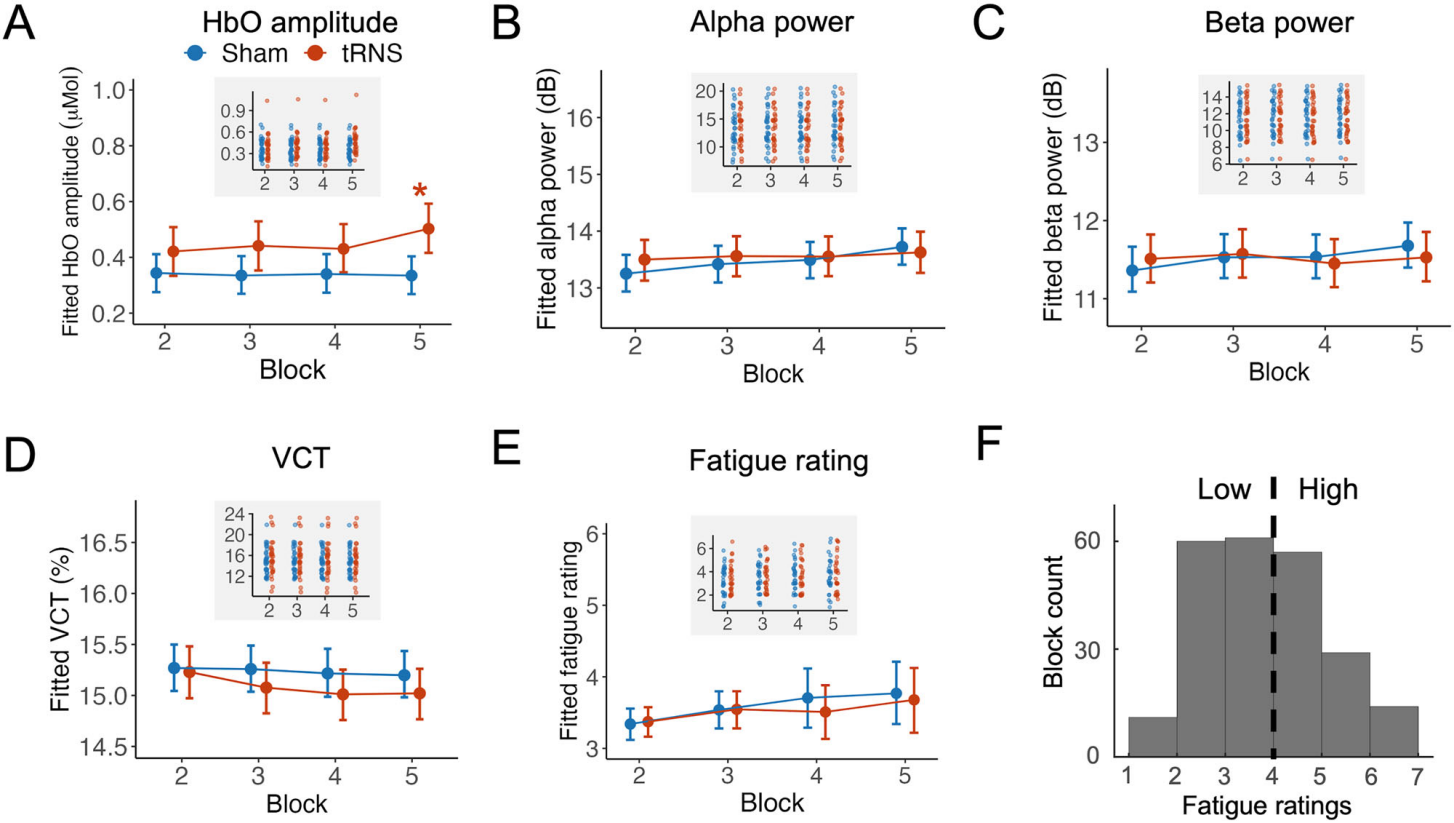
tRNS effects without considering fatigue. **A** tRNS increased HbO amplitude in the fifth block compared to sham stimulation. The asterisk (*) indicates a posterior probability exceeding 97.5%, with a 95% HPD interval that does not include zero, providing credible evidence (i.e., statistically significant under Bayesian criteria). The inset plot with a gray background displays fitted individual data points. **B** tRNS did not affect prestimulus alpha power. **C** tRNS did not affect prestimulus beta power. **D** tRNS did not affect VCT. **E** Fatigue ratings were unaffected by tRNS compared to sham stimulation, confirming fatigue as an independent variable for categorization. **F** Given the independence of fatigue ratings from stimulation conditions, fatigue ratings across blocks were categorized into two groups: low fatigue (<4) and high fatigue 4 ≥ 4).

We first analyzed the effects of tRNS on HbO amplitude, prestimulus alpha and beta power, and VCT without considering fatigue states, using Bayesian linear mixed models (see details in Methods). Evidence supporting a hypothesis was considered credible and interpreted as statistically significant under Bayesian criteria if the posterior probability (*Pr*) exceeded 97.5 and the 95% highest posterior density (HPD) interval did not include zero. Compared to the sham condition, tRNS increased HbO amplitude only in the 5th block (Fig. 3A; *E*(*μ*_Sham-tRNS, Block5_) = −0.169, HPD = [−0.278, −0.063], Pr(*μ*_Sham-tRNS, Block5_ < 0) = 0.999; see complete statistics in Supplementary Table 1). However, tRNS did not modulate prestimulus alpha power (Fig. 3B; all *Pr*s < 0.975; see Supplementary Table 2), prestimulus beta power (Fig. 3C; all *Pr*s < 0.975; see Supplementary Table 3), or VCT (Fig. 3D; all *Pr*s < 0.975; see Supplementary Table 4).

Considering accumulating evidence that brain states influence the effects of tES^26^, we included fatigue as an interaction factor in subsequent analyses. Before examining state-dependent effects, we verified that fatigue ratings were not influenced by tRNS. The results confirmed that tRNS did not affect fatigue ratings compared to the sham condition (Fig. 3E; all *Pr*s < 0.975; see Supplementary Table 5), affirming the independence of fatigue from the stimulation conditions. Consequently, data samples—including fNIRS HbO amplitude, EEG power in different frequency bands, and VCT—were categorized into low and high fatigue groups using a threshold of 4 on the 7-point scale (Fig. 3F), distinguishing low fatigue (<4) from high fatigue (≥4), consistent with our prior study^29^.

### tRNS increased HbO, decreased alpha/beta power, and reduced VCT under low fatigue

**Fig. 4:**
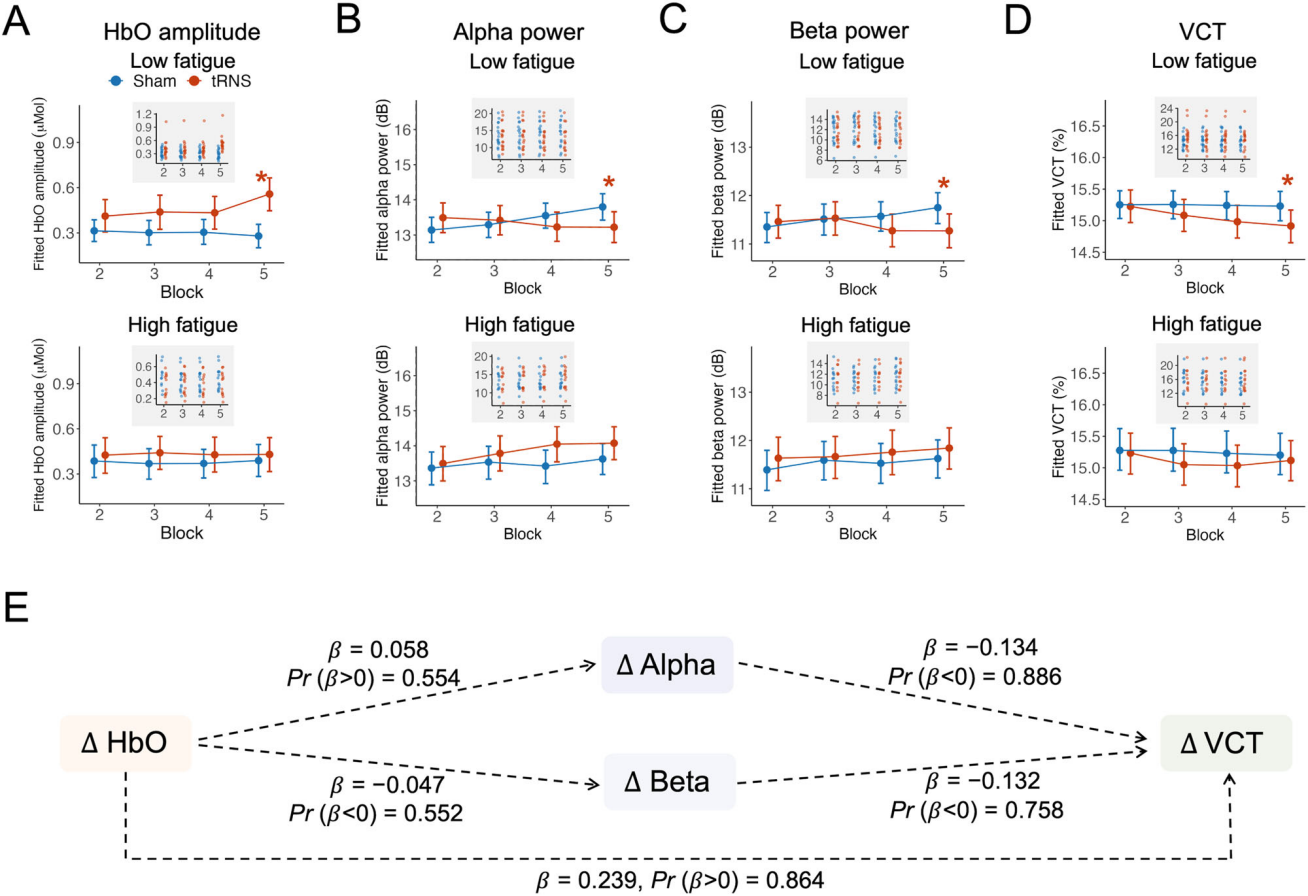
State-dependent effects of tRNS on neural and behavioral measures, and exploratory associations among their changes. **A–D** Under low fatigue, tRNS significantly increased HbO amplitude (**A**), decreased prestimulus alpha (**B**) and beta (**C**) power, and improved VCT performance (**D**) in Block 5 compared to sham stimulation. No stimulation effects were observed under high fatigue across any block. Asterisks (*) indicate posterior probabilities (*Pr*) exceeding 97.5% with 95% HPD excluding zero, indicating strong evidence for a tRNS effect. The inset plot with a gray background displays fitted individual data points. **E** Exploratory post hoc analysis of stimulation-induced changes (Δ = tRNS–sham) in Block 5 under low fatigue. Dashed arrows represent Bayesian regression slopes for each pairwise association. While the group-level effects were robust, the between-participant associations among ΔHbO, ΔAlpha/ΔBeta, and ΔVCT were weak and statistically inconclusive. Mediation models showed similarly uncertain indirect effects (see Results), suggesting that the neural and behavioral responses to tRNS, although parallel in timing, did not scale linearly across individuals.

We next analyzed the effects of tRNS while considering fatigue as an interaction factor, using Bayesian linear mixed models. In the 5th block under low fatigue, compared to the sham condition, tRNS significantly increased HbO amplitude (Fig. 4A; *E* (*μ*_Sham-tRNS_) = −0.276, HPD = [−0.405, −0.137], *Pr* (*μ*_Sham-tRNS_ < 0) = 1; see Supplementary Table 6), decreased prestimulus alpha power (Fig. 4B; *E* (*μ*_Sham-tRNS_) = 0.582, HPD = [0.034, 1.162], *Pr*(*μ*_Sham-tRNS_ > 0) = 0.977; see Supplementary Table 7), decreased prestimulus beta power (Fig. 4C; *E* (*μ*_Sham-tRNS_) = 0.482, HPD = [0.047, 0.927], *Pr* (*μ*_Sham-tRNS_ > 0) = 0.983; see Supplementary Table 8), and reduced VCT (Fig. 4D; *E* (*μ*_Sham-tRNS_) = 0.307, HPD = [0.001, 0.603], *Pr* (*μ*_Sham-tRNS_ > 0) = 0.978; see Supplementary Table 9). In contrast, tRNS did not have significant effects in other blocks or under high fatigue (Fig. 4). Similar patterns were not found in prestimulus delta, theta, and gamma power (Supplementary Fig. 2). For descriptive purposes, we also provide raw EEG power spectra for tRNS and sham conditions in Block 5 (Supplementary Fig. 3). These spectra show the overall frequency profile across conditions but should not be interpreted as a statistical test of the effects reported in Fig. 4, as they are based on raw (non-modeled) power.

To further examine whether stimulation-induced neural changes co-varied with behavioral improvements, we tested whether ΔHbO (tRNS−Sham), ΔAlpha, and ΔBeta were associated with ΔVCT across participants. These associations were modeled across all blocks and fatigue states (see Methods), maintaining the factorial structure `used above, with simple slopes (*β*) extracted for Block 5 under low fatigue where group-level effects were strongest. As shown in Fig. 4E, we observed weak and inconclusive evidence for positive associations between ΔHbO and ΔVCT (*β* = 0.239, HPD = [–0.230, 0.864], *Pr*(*β* > 0) = 0.864), and between ΔHbO and ΔAlpha (*β* = 0.058, HPD = [–0.244, 0.444], *Pr*(*β* > 0) = 0.554). Associations between ΔHbO and ΔBeta were similarly small and uncertain (*β* = –0.047, HPD = [–0.407, 0.308], *Pr*(*β* < 0) = 0.552). The slopes from ΔAlpha to ΔVCT (*β* = –0.134, HPD = [–0.460, 0.152], *Pr*(*β* < 0) = 0.886) and from ΔBeta to ΔVCT (*β* = –0.132, HPD = [–0.453, 0.249], *Pr*(*β* < 0) = 0.758) were negative in direction but did not provide credible evidence. These results suggest that although neural and behavioral responses to tRNS occurred in parallel, their magnitudes did not linearly covary across individuals under peak-effect conditions. Consistently, exploratory mediation analyses revealed inconclusive indirect effects for both alpha (*β* = –0.065, HPD = [–1.882, 1.454], *Pr*(*β* < 0) = 0.586) and beta (*β* = 0.002, HPD = [–1.304, 1.475], *Pr*(*β* > 0) = 0.507), further indicating weak evidence for a structured mediation pathway.

### Fatigue modulates neural responsiveness to tRNS in prestimulus oscillations

To clarify how outcomes vary across fatigue levels within each stimulation condition, we compared neural and behavioral measures between low- and high-fatigue states, separately for tRNS and sham, using contrasts derived from the same Bayesian linear mixed-effects model of Fig. 4.

**Fig. 5:**
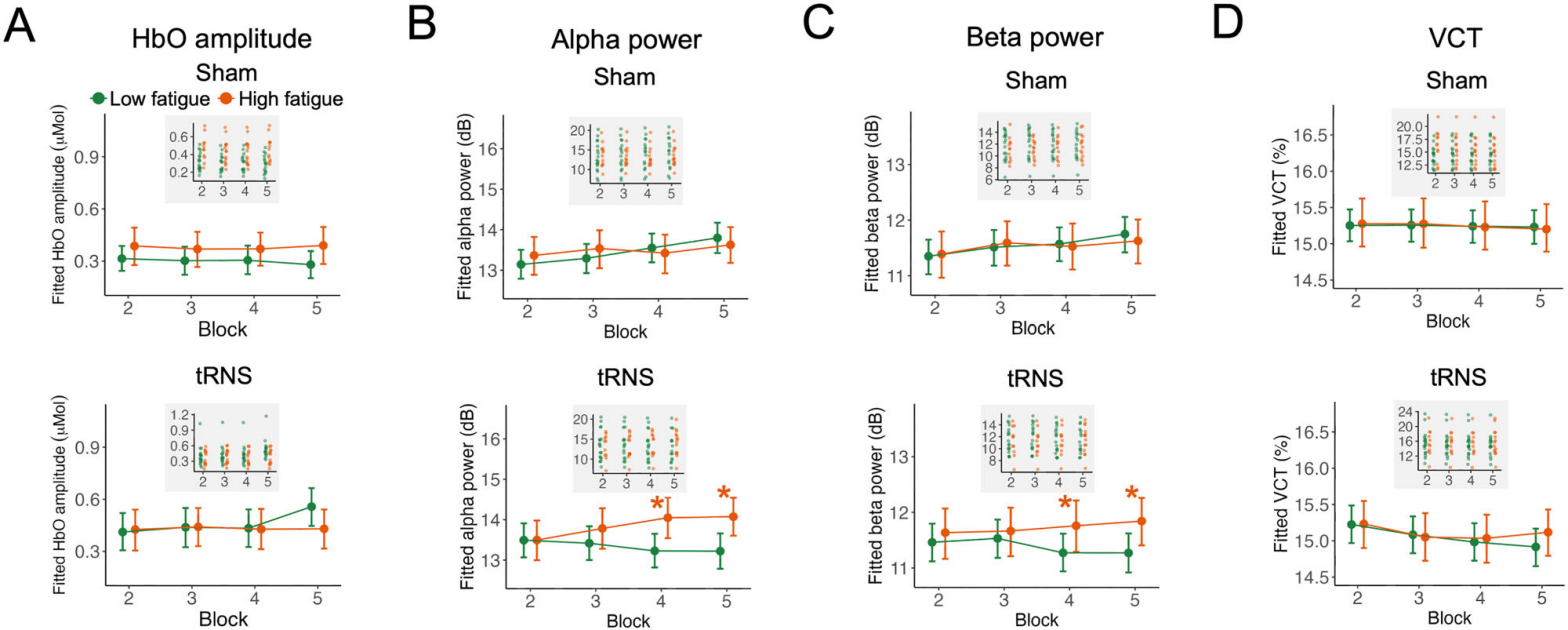
Fatigue modulates the neural effects of tRNS on prestimulus oscillatory power but not hemodynamic or behavioral outcomes. **A** Despite the visually larger HbO amplitude in low fatigue compared to high fatigue in Block 5 for tRNS (*Pr* < 97.5%, see Supplementary Table 10), no significant differences in HbO amplitude were observed between fatigue states under either condition. The inset plot with a gray background displays fitted individual data points. **B** Under tRNS, prestimulus alpha power was significantly lower in low-fatigue compared to high-fatigue states in Blocks 4 and 5; no such difference was found under sham. **C** Beta power showed a similar pattern, with significant fatigue-related suppression only under tRNS. **D** VCT did not differ between fatigue states in either condition. These results suggest that fatigue modulates neural responsiveness to tRNS in the oscillatory domain, while hemodynamic and behavioral measures remain stable.

For HbO amplitude and VCT, we found no significant differences between low- and high-fatigue states under either tRNS or sham, indicating that fatigue alone did not significantly alter hemodynamic or behavioral measures (Fig. 5A, D).

In contrast, prestimulus alpha and beta power were significantly lower under low fatigue than under high fatigue—but only during tRNS, not sham (Fig. 5B, C). These differences emerged specifically in Blocks 4 and 5, which are also the periods where tRNS effects were strongest overall (see Fig. 4). This pattern suggests that fatigue modulates the brain’s responsiveness to stimulation, rather than oscillatory baselines in the absence of stimulation. Full model statistics are provided in Supplementary Table 10–13.

### State-dependent sensitivity analysis highlights alpha as the key perceptual band

**Fig. 6:**
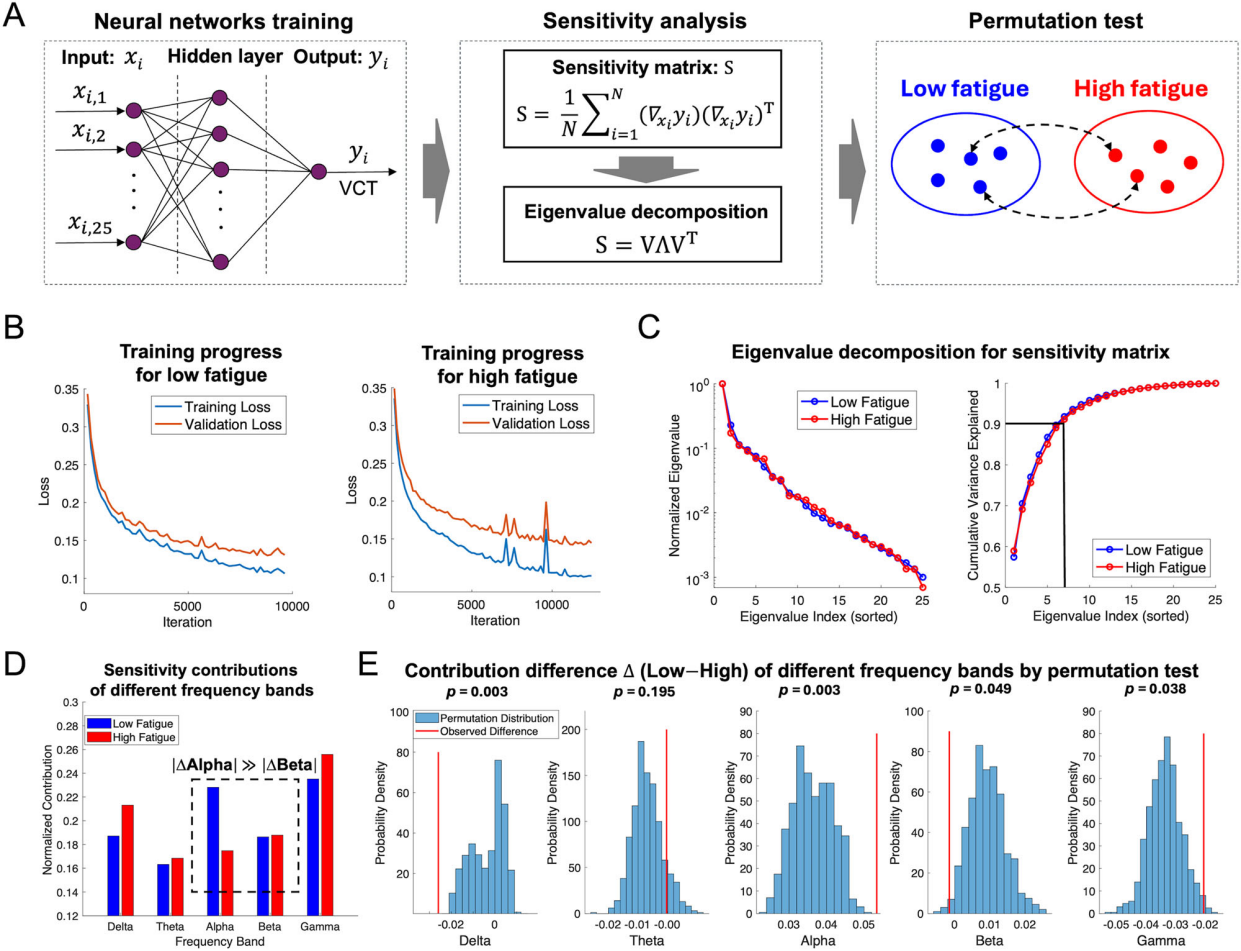
Sensitivity analysis links fatigue state to frequency-specific perceptual weighting. **A** Analysis pipeline. A feed-forward neural network (25 inputs = 5 bands × 5 electrodes; one hidden layer; single VCT output) was trained separately on low- and high-fatigue trials from the sham condition. From the trained weights we formed the sensitivity matrix S and obtained band-wise contributions by eigen-decomposition. A permutation test (10000 shuffles of fatigue labels) assessed whether the observed Low–High difference for each band exceeded chance. **B** Learning curves for the two networks (8:2 train/validation split, early stopping). **C** Eigen spectrum of S (left) and cumulative variance (right). The first seven eigen-vectors (vertical line) explain ≥ 90% of the variance and were used to compute contributions. **D** Normalized contributions of the five bands. Blue = Low-fatigue, red = High-fatigue. A dashed box highlights that the Low–High gap for Alpha (ΔAlpha) greatly exceeds that for Beta (ΔBeta). **E** Permutation distributions of Δ = Low–High for each band. Red line = observed Δ. Two-tailed *p*-values (FDR-corrected) appear above each panel. ΔDelta and ΔAlpha are significant; smaller but significant effects appear for Beta and Gamma, whereas Theta shows no state dependence.

Building on the observation that tRNS modulated alpha and beta power only under low fatigue (Fig. 4), and that these bands were more responsive to stimulation in that state (Fig. 5), we next asked whether such state-dependent modulation reflects differences in their functional relevance for perception. To clarify the frequency-specific mechanisms linking prestimulus oscillatory activity to perceptual performance, we conducted a sensitivity analysis comparing how EEG band power predicted VCT under low and high fatigue. This analysis enabled us to identify which frequency bands contribute most to perceptual variability across states, independent of tRNS. We trained two separate neural network models using data merely from the sham group—one for low-fatigue trials and one for high-fatigue trials—to capture potential nonlinear relationships between single-trial EEG features and VCT (Fig. 6A). Each model received 25 input features (5 frequency bands × 5 occipital electrodes) and was trained to predict VCT across 6640 low-fatigue and 4960 high-fatigue samples, respectively.

To prevent overfitting, the data were split into training and validation sets (8:2 ratio), and early stopping was employed during training. Both networks showed stable convergence of loss across iterations (Fig. 6B). We computed the sensitivity matrix from each trained network and performed eigenvalue decomposition to quantify the contribution of each band to VCT prediction. As shown in Fig. 6C, the first seven eigenvectors captured over 90% of the total variance, and were used to compute sensitivity contributions.

Figure 6D presents the normalized contributions of each frequency band in low and high fatigue states. While gamma showed the highest raw contribution overall, we observed a notably larger contribution of the alpha band under low fatigue (0.227) compared to high fatigue (0.174). In contrast, beta showed a small reduction in contribution from high to low fatigue (0.188 to 0.186), suggesting a relatively modest state-dependent change.

To assess whether the observed differences in frequency-band contributions exceeded what could be expected by chance, we performed permutation tests by randomly shuffling fatigue labels 10000 times (Fig. 6E). The resulting permutation distributions were slightly shifted away from zero due to the non-linear model structure and unequal sample sizes, producing non-zero baselines. However, this shift reflects the expected contribution difference under random relabeling, and any significant deviation from this baseline still indicates a reliable effect of fatigue. The alpha band showed a significantly greater contribution under low fatigue (*p* = 0.003, FDR-corrected), exceeding its elevated chance level and supporting its state-dependent functional relevance. Delta showed a significant negative difference (*p* = 0.003), indicating increased contribution under high fatigue. Smaller but significant effects were also observed for beta (*p* = 0.049) and gamma (*p* = 0.038), both showing reduced contributions in low-fatigue trials relative to their respective chance baselines. Theta showed no significant difference between states (*p* = 0.195).

Together, these results suggest that both alpha and beta bands may contribute to perceptual performance under low fatigue, especially given that both were suppressed by tRNS (Fig. 4) and showed greater responsiveness in that state (Fig. 5). However, our sensitivity analysis (Fig. 6) provides critical evidence that the perceptual system relies more heavily on alpha power: the Low–High sensitivity shift for alpha was not only statistically significant, but also substantially larger in magnitude than that for beta. While beta showed a small but reliable reduction in contribution under low fatigue, the alpha band exhibited the most prominent state-dependent increase. These findings imply that although tRNS modulates both alpha and beta activity in low fatigue, the resulting improvement in VCT is likely driven predominantly by changes in alpha-band dynamics.

## Discussion

This study aimed to clarify the causal relationship between prestimulus low-frequency oscillations and visual perception by using tRNS to directly modulate neural activity. We found that tRNS, particularly under low fatigue, led to increased cortical excitability (indexed by fNIRS HbO amplitude), suppressed subsequent prestimulus alpha and beta power, and improved visual contrast sensitivity (reduced VCT; Figs. 3 & 4). These effects were absent under high fatigue, indicating strong state dependence. Further analyses revealed two complementary mechanisms underlying this causal link: first, low-fatigue states enhanced the brain’s responsiveness to stimulation, enabling modulation of alpha and beta rhythms (Fig. 5); second, sensitivity analysis showed that alpha oscillations, in particular, became more behaviorally relevant under low fatigue, while beta showed a smaller effect (Fig. 6). Taken together, these results provide converging evidence that tRNS can enhance visual perception by modulating prestimulus alpha and beta power, but only when the brain is in a receptive state and the targeted oscillations are functionally linked to behavior, establishing a state-dependent causal pathway between neural oscillations and perception.

We first analyzed the effects of tRNS without considering fatigue states. The stimulation effects were minimal compared to the state-dependent effects shown later, highlighting the potential confounding role of internal states. This contrast, attributable to fatigue states—or brain states in a broader sense—may be one of the main reasons for inconsistent results in existing tES studies^26,30–32^, and it aligns with growing evidence that brain-state variables critically determine stimulation efficacy^26^. We then analyzed the effects of tRNS on fatigue itself and found no significant change in subjective ratings across stimulation conditions. These null effects suggest that fatigue, though influential, was not itself modulated by stimulation. This dissociation supports the idea that fatigue and oscillatory mechanisms operate through partially independent processes^33^, although both shape perceptual readiness.

To resolve the ambiguity observed in the global model, we next analyzed tRNS effects while explicitly accounting for fatigue states. As shown in Fig. 4, tRNS effects on neural and behavioral measures were significant only in low fatigue, particularly in Block 5. Specifically, tRNS increased HbO amplitude, suppressed alpha and beta power, and improved VCT under low fatigue, but had no such effects under high fatigue. These results reinforce the idea that tRNS effects are strongly state-dependent, with fatigue modulating neural responsiveness. The block specificity of these effects, concentrated in Block 5, further suggests that stimulation efficacy accumulates over time, possibly due to delayed engagement of plasticity mechanisms^34^.

To examine whether fatigue itself influenced neural dynamics independently of stimulation, we also compared low- and high-fatigue states within each stimulation condition (Fig. 5). Interestingly, we found no significant differences in HbO or VCT, but prestimulus alpha and beta power were significantly lower under low fatigue only in the tRNS condition. This pattern demonstrates that fatigue does not broadly shift baseline activity but rather modulates the brain’s responsiveness to stimulation. The emergence of this effect in both Block 4 and Block 5 suggests that neural excitability under low fatigue begins to support stimulation-induced modulation before behavioral changes fully manifest. This finding supports the idea that fatigue acts as a gate, controlling whether tRNS can effectively shape ongoing neural rhythms.

The state-dependent effects of tRNS suggest a causal role of prestimulus alpha and beta oscillation power in visual perception. This interpretation is supported by several core principles of causal inference^16,35,36^. First, the relationship exhibits temporal precedence (the cause must occur before the effect), as our manipulation of brain state via tRNS and the resulting changes in cortical excitability (HbO) and neural oscillations (EEG) all preceded the observed behavioral outcome (VCT). Second, we observed covariation (the effect changes systematically with the cause), as tRNS led to consistent changes in HbO amplitude, alpha/beta power, and visual perception specifically under low fatigue. Although these covariations were evident at the group level, exploratory correlation and mediation analyses of stimulation-induced changes across individuals revealed weak and inconclusive associations among HbO, oscillatory power, and VCT (Fig. 4E). This dissociation likely reflects a combination of inter-individual variability, measurement noise, and non-linear dose–response dynamics of tRNS^37–39^. Third, the findings exhibit biological plausibility (a well-supported mechanism links the cause and effect), given that reduced alpha/beta power reflects increased excitability and is known to improve sensory processing^1^. Together, these properties strengthen the causal interpretation of our findings. The possibility that other frequency bands might contribute was ruled out through additional analysis of delta, theta, and gamma power (Supplementary Fig. 2).

Given that tRNS modulated alpha and beta power only under low fatigue, we next asked whether the perceptual system also relies differently on these frequencies across states. The sensitivity analysis (Fig. 6) revealed that alpha-band power showed the largest increase in contribution to predicting VCT under low fatigue, indicating that alpha oscillations become especially important for perception in a more alert state. Although beta also showed a significant state-dependent shift, the magnitude of its change was much smaller, suggesting a limited role in perceptual gating. In contrast, delta and gamma contributions increased under high fatigue, pointing to a reallocation of perceptual sensitivity away from the frequencies most modulated by tRNS. These results suggest that internal brain state dynamically shifts the spectral basis of perception: in the low-fatigue state, perception relies more on alpha, while in high fatigue, it becomes more dependent on lower (delta) or higher (gamma) frequencies. This frequency-specific sensitivity pattern provides a crucial interpretive bridge between the neural and behavioral results shown in Figs. 4 and 5. In low-fatigue blocks, tRNS suppresses both alpha and beta power (Fig. 4), and Fig. 5 shows that these bands are more responsive to stimulation in this state. However, only alpha band shows a significant increase in perceptual relevance under low fatigue (Fig. 6), consistent with a large body of research predominantly linking alpha activity to fluctuations in visual perception^1,40,41^. Taken together, these results suggest that tRNS is effective only when it modulates frequency bands that are both dynamically responsive and behaviorally relevant, conditions met uniquely in the low-fatigue state through alpha-band engagement. While our study focused on tRNS, it is worth noting that other techniques, such as transcranial magnetic stimulation (TMS), have also been used to modulate alpha and beta oscillations and link them to behavior^42^. Our findings complement this broader body of work, supporting the causal role of low-frequency rhythms in perception.

Our findings have significant implications for developing interventions aimed at enhancing sensory processing and cognitive functions, particularly for individuals with perceptual or attentional disorders. Disorders such as amblyopia, attention deficit-hyperactivity disorder (ADHD), and visual neglect are associated with abnormalities in neural oscillations and perceptual deficits^43,44^. By demonstrating that modulating prestimulus alpha and beta power can causally influence visual perception, our study suggests that tRNS could serve as a non-invasive neuromodulation technique for therapeutic interventions. tRNS has been shown to enhance cortical excitability and improve cognitive functions without discomfort^19^. Previous research indicates that tRNS can facilitate perceptual learning in amblyopia patients^45^ and improve attentional performance in individuals with ADHD^46,47^, etc. Considering the state-dependent nature of tRNS effects, personalized interventions that account for individual brain states, such as fatigue levels, could optimize efficacy^26^. Future studies should explore the clinical applications of tRNS in these populations, potentially leading to novel treatments for perceptual and attentional disorders.

It should be noted that the prestimulus alpha and beta oscillations we investigated were “online” with visual perceptual behavior (VCT) but “offline” with respect to tRNS application. In contrast, the fNIRS HbO signal captured hemodynamic changes during stimulation. While this dissociation in timing might suggest a sequential relationship, we cannot directly conclude that neurovascular responses precede neural oscillatory changes. It remains possible that EEG changes also occurred during stimulation but were not measurable due to tRNS-related artifacts. Moreover, both the online and offline effects exhibited an accumulation effect; that is, tRNS effects became noticeable after several blocks of stimulation. Similar accumulation effects were observed in our previous study^29^ and can be echoed by studies showing that incremental changes due to neural stimulation may accumulate over time, leading to a threshold-crossing effect^34^.

There are some limitations in our study. First, while subjective ratings provided a “gold standard” of mental fatigue, they are still relatively subjective compared to some objective physiological measures, such as pupil size^48^. Therefore, future work could employ such measures to quantify brain states when studying state-dependent effects of tES. Second, although fNIRS and EEG were used to capture temporally distinct stages of the stimulation-perception pathway, their non-overlapping acquisition windows prevented us from testing their trial-level coupling directly. This design choice was necessary to avoid EEG artifacts during stimulation, but it limited our ability to establish a fine-grained mechanistic relationship. Future studies using concurrent EEG-fNIRS recordings or improved artifact mitigation could help directly validate the neurovascular-oscillatory link. Third, we mainly collected and analyzed localized neural activity driven by our hypothesis. It would be beneficial to also analyze neural activity in other brain areas, which could provide a more comprehensive understanding of the neural mechanisms involved.

In conclusion, our study supports the causal relationship between prestimulus alpha and beta power and visual perception by combining fNIRS/EEG recordings and tRNS. The establishment of this causal link not only advances our understanding of the neural mechanisms underlying visual perception but also has broader implications for developing interventions aimed at enhancing sensory processing and cognitive functions.

## Methods

### Participants

This study included 29 participants (15 females; mean age, 22.7 ± 1.9 years) after excluding nine individuals due to misunderstanding of instructions, excessive EEG or fNIRS artifacts, or discomfort during stimulation. The original sample size of 38 was consistent with our previous studies^12,29^ to yield more comparable results. All participants were right-handed, on-campus students with normal or corrected-to-normal vision. Exclusion criteria were stringent, encompassing left-handedness; a history of seizures or head injuries; current use of psychotropic medication; presence of metal implants in the head; implanted electronic devices; psychiatric disorders; tinnitus; or participation in other neuromodulation experiments within the last three months. Prior to the experiment, participants were instructed to rest adequately and abstain from caffeine or alcohol. Informed consent was obtained from all participants. The study was approved by the local ethics committee at Shenzhen University. All ethical regulations relevant to human research participants were followed.

### Experimental Design

We employed a visual detection task adapted from previous studies^8,9^, implemented in MATLAB (MathWorks, Natick, MA) using Psychophysics Toolbox 3. The experiment was conducted in a dark, acoustically isolated chamber. Visual stimuli consisted of near-threshold Gabor patches displayed on a 21-inch LCD monitor with a 100 Hz refresh rate, positioned 60 cm from the participants. These patches, tilted at 10 degrees and subtending a visual angle of 0.75 degrees, appeared on either side of a central fixation dot for 0.2 s in 60% of the trials. Participants responded by pressing buttons after a 0.4 s delay when the fixation dot changed to a question mark. Feedback was provided by displaying a green (correct) or red (incorrect) dot for 0.2 s. Each trial was followed by a blank screen interval for blinking and an inter-stimulus interval (ISI) of 1.8–2.4 s (Fig. 2A).

The experiment consisted of five blocks, each beginning with a 5 min resting-state period during which fNIRS signals were recorded. tRNS was applied during rest in Blocks 2–5, but not in Block 1. Following the rest period, participants completed a visual detection task during which EEG data were collected (Fig. 2A). Block 1, which involved no stimulation, served as the baseline for subsequent statistical comparisons. Each block included 80 trials, with stimulus contrast adjusted dynamically to maintain a 50% hit rate using the adaptive staircase procedure QUEST^49^. The QUEST algorithm was run continuously throughout the experiment, updating its priors based on the results from the preceding block (Supplementary Fig. 1A). We fitted the stimulus contrast values within each block using a cumulative Gaussian function and estimated the VCT as the mean of this function^29^.

### Transcranial random noise stimulation

tRNS was administered using a high-definition transcranial electrical stimulator (Soterix Medical) with an M × N 33-channel configuration. Four 12 mm-diameter Ag/AgCl ring electrodes filled with conductive gel were placed over CPz, P5, P6, and Oz (Fig. 2B). The tRNS stimulation frequencies ranged from 100 to 640 Hz in the high-frequency band^19^, with a maximum current intensity of 1 mA (99% of amplitude values between –0.5 and 0.5 mA), as shown in Supplementary Fig. 1B.

The study followed a sham-controlled, single-blind, within-subject design with two stimulation conditions: sham and tRNS. Each participant underwent both conditions, with a 3–7 day interval between sessions. All stimulation conditions included a 10 s fade-in/fade-out period. The sham stimulation lasted only for the first 30 s, while tRNS was applied during the resting periods between blocks (Fig. 2A).

Participants provided self-reported fatigue ratings on a 7-point scale before and after each block. A rating of 1 indicated no fatigue, 4 moderate fatigue, and 7 extreme fatigue. The average score represented the prevalent fatigue state for each block. Data samples including VCT, EEG, and fNIRS features were classified based on these fatigue ratings, using a threshold of 4 to distinguish between low fatigue (<4) and high fatigue (≥4)^50,51^. This division facilitates the interpretability of models with interaction effects and is consistent with our prior study^29^. Consequently, this measure was represented within each stimulation condition and participant, allowing within-subject comparisons. It was also based on the recognized interplay between brain states and stimulation effects; specifically, conditions like arousal and sleepiness, which correlate with fatigue, significantly influence responses to electrical stimulation^23,24,26,52,53^. Note that in the histogram of Fig. 3F, the first five bars include values from the lower edge up to but excluding the upper edge, while the sixth bar includes values up to and including the upper edge. Consequently, we hypothesized state-dependent tRNS effects during the experiment—that is, tRNS effects would vary under different levels of fatigue.

### EEG data collection, preprocessing, and computation of alpha/beta power

EEG signals were recorded using a 64-channel electrode system (Easycap) and an EEG amplifier (BrainAmp, Brain Products GmbH, Germany), following the international 10-10 system. Electrodes were positioned over occipital channels (O1, O2, PO3, PO4, POz), with FCz as the reference electrode. Impedances were kept below 10 kΩ. The raw EEG data were preprocessed using the EEGLAB toolbox^54^, including resampling to 250 Hz and band-pass FIR filtering from 0.1 to 80 Hz. Data were then segmented into epochs from –1800 to 1500 ms relative to stimulus onset, followed by visual artifact removal.

To compute prestimulus alpha and beta power, we estimated the power spectral density (PSD) of prestimulus EEG signals from each occipital electrode to quantify power within five specific frequency bands: delta (0.5–4 Hz), theta (4–8 Hz), alpha (8–13 Hz), beta (13–30 Hz), and gamma (30–45 Hz). We limited the gamma band to 45 Hz to avoid contamination from 50 Hz line noise and potential artifacts at higher frequencies. The PSD was estimated using Welch’s method with a Hamming window length of 200 samples, 50% overlap (100 samples), and an FFT length of 256 points, balancing spectral resolution and variance reduction. We calculated the power in each frequency band by averaging the PSD over the respective frequency ranges and converted these values to decibels (dB) to facilitate comparison across bands and electrodes. All computations were performed using MATLAB R2023b with built-in functions (pwelch and bandpower), ensuring consistency and reproducibility.

### fNIRS data collection, preprocessing, and computation of HbO amplitude

fNIRS data were collected using a NIRSport2 system (NIRx Medical Technologies, LLC, New York, NY) at a sampling rate of 10.1725 Hz, employing wavelengths of 760 nm and 850 nm. The setup included six source probes and six detector probes, inserted into holders on a cap arranged according to the international 10-5 system (sources at PPOz, PPO3, PO1h, POO3, POO4, PPO4; detectors at PPO1, POOz, PO2h, PPO2, POO8, POO7), covering the occipital lobe through a network of 21 channels (Fig. 2B). The source-detector distances varied around an average of 32.743 ± 8.510 mm, optimized to accommodate simultaneous EEG and tRNS setups. To minimize external light interference, a dark over-cap was used. Changes in light attenuation were analyzed using the modified Beer-Lambert law to derive relative changes in the concentrations of HbO and deoxyhemoglobin (HbR). The HbO and HbR signals were high-pass filtered at 0.015 Hz and low-pass filtered at 0.1 Hz to eliminate high-frequency physiological noise and low-frequency baseline drifts. An artifact rejection threshold was established at the mean concentration plus or minus two standard deviations. Data collected at the beginning and end of measurements were discarded if HbO levels exceeded this threshold, adjusting for movement artifacts. After preprocessing, the fNIRS data reflected the relative changes of HbO and HbR in micromoles (μMol). To assess global variations during the resting periods, we calculated the mean time series of relative changes across all channels to derive the global signal in the occipital lobe. This averaging process enhanced the signal-to-noise ratio (SNR) and mitigated potential effects arising from variable channel distances. The amplitude of the HbO signal was quantified using the standard deviation of this averaged time series^55^.

### Statistical analysis of tRNS effects

To evaluate the potential causal role of prestimulus alpha and beta power on VCT, we first compared tRNS effects on HbO amplitude, prestimulus alpha and beta power, and VCT across the two stimulation conditions (sham and tRNS) using Bayesian linear mixed models, implemented via the Stan modeling language^56^ and the brms package^57^. The Bayesian approach was adopted for its benefits in resolving non-convergence problems and facilitating the fitting of maximal varying effect structures^58^. We then examined whether different stimulation conditions influenced fatigue ratings, which could potentially confound the categorization of low/high fatigue^59^. Finally, we analyzed how fNIRS HbO amplitude, EEG prestimulus power in different frequency bands, and VCT varied across blocks in the different stimulation conditions under different fatigue levels (low/high). Baseline measurements from Block 1 were included in all models as covariates, and the sham condition at Block 1 always served as the reference level for categorical contrasts. This approach allowed us to evaluate the effects of stimulation and fatigue relative to a common baseline across participants. The results were evaluated by calculating the posterior probability of the contrast difference, *δ*, between estimated parameters corresponding to tRNS and sham conditions. Models hypothesized that *δ* ≠ 0. Evidence supporting a hypothesis was considered credible and interpreted as statistically significant under Bayesian criteria if the posterior probability (*Pr*) exceeded 97.5% and the 95% HPD\ interval did not include zero; otherwise, the evidence was considered inconclusive^60,61^. Four sampling chains were run for 2000 iterations, with the first 1000 as the warm-up phase. The default priors of the brms package were applied. The specific models employed were as follows:

1. tRNS effects on HbO amplitude, prestimulus alpha and beta power, VCT, and fatigue ratings (Fig. 3)
  HbO amplitude, prestimulus alpha and beta power, VCT, and fatigue ratings were modeled as a function of baseline measurements, stimulation condition, block, and the interaction between stimulation and block, with individual participants as random effects. For the random effects part, we did not include the interaction of block due to increased complexity and implementation issues (i.e., the model fit badly). The model syntax was:
  $$	{HbO\; amplitude}/{prestimulus\; alpha\; and\; beta\; power}/{VCT}/{fatigue\; ratings} \sim 1\\ 	+{Baseline}+{Condition}\times {Block}+(1+{Condition}|{Participant})$$
2. State-dependent tRNS effects depending on fatigue level (Fig. 4A–D and Fig. 5):
  HbO amplitude, prestimulus power, and VCT were modeled to analyze the impact of fatigue level, the stimulation condition, block, and the interaction between stimulation and block. For the random effects part, we did not include the interaction of block due to increased complexity and implementation issues. The models were formulated as:
  $$	{fNIRS}/{EEG}/{VCT} \sim 1+{Baseline}+{Fatigue\; level}\times {Condition}\times {Block}\\ 	+(1+{Fatigue\; level}\times {Condition}|{Participant})$$
3. Associations among stimulation-induced changes (Fig. 4E):
  To examine whether stimulation-induced changes in haemodynamic response (ΔHbO; tRNS−sham), prestimulus oscillatory power (ΔAlpha and ΔBeta), and visual performance (ΔVCT) co-varied across participants, we fitted a series of Bayesian linear mixed-effects models. These models maintained consistency with the main factorial analysis (Fig. 4A–D) by incorporating all blocks and fatigue levels. Each model included the interaction of the predictor with fatigue level and block. Random slopes were specified for fatigue level but not for block, as including block as a random slope led to poor model convergence. The model syntax was:
  - ΔVCT ~ 1 + ΔHbO × *Fatigue level* × *Block* + (1 + *Fatigue level* | *Participant*)
  - ΔVCT ~ 1 + ΔAlpha/Beta × *Fatigue level* × *Block* + (1 + *Fatigue level* | *Participant*)
  - ΔAlpha/Beta ~ 1 + ΔHbO × *Fatigue level* × *Block* + (1 + *Fatigue level* | *Participant*)
4. Mediation analysis in Block 5, low-fatigue state:

To further test whether oscillatory changes mediated the effect of ΔHbO on ΔVCT, we conducted a Bayesian mediation analysis restricted to Block 5 and low-fatigue trials, where group-level effects were strongest. The mediation model consisted of two linked regression equations: one regressing ΔAlpha/Beta on ΔHbO (the mediator model), and the other regressing ΔVCT on both ΔHbO and ΔAlpha/Beta (the outcome model). Random intercepts were included for each participant, and residual correlations between equations were set to zero. The indirect effect was computed as the product of the ΔHbO → ΔAlpha and ΔBeta → ΔVCT paths. Posterior medians, 95% HPD, and directional probabilities were extracted from the posterior samples for indirect, direct, and total effects.

### Sensitivity analysis

To compare how EEG power across different frequency bands contributes to VCT under varying fatigue states, we trained neural network models to predict single-trial VCT from EEG power in the sham group, which served as a baseline independent of tRNS effects. After training, we computed the sensitivity matrix and applied eigenvalue decomposition to quantify the relative importance of each frequency band. Finally, we conducted permutation tests to examine whether the state-dependent effects of tRNS could be explained by differences in the perceptual relevance of each frequency band between low- and high-fatigue states.

We chose neural network models to capture potential nonlinear relationships between EEG power and VCT. Two separate models were trained using data from the sham group under low and high fatigue, respectively. Both models had identical architectures and hyperparameters: one hidden layer with 64 units, a batch size of 32, a maximum of 200 epochs, a learning rate of 0.001, an optimizer of Adam, and the ReLU activation function. The input *x*_*i*_ consisted of 25 features representing EEG power across five frequency bands and 5 electrodes (denoted as *x*_*i*,1_, *x*_*i*,2_,…, *x*_*i*,25_, in Fig. 6A), and the output *y*_*i*_ was the single-trial VCT. Since VCT was measured every 40 trials within a block, each group of 40 samples shared the same VCT value. The datasets comprised 6640 samples for low fatigue and 4960 samples for high fatigue. To prevent overfitting, we split each dataset into training and validation subsets in an 8:2 ratio and utilized early stopping with a patience of five epochs. For the low fatigue model, training stopped after 58 epochs (iteration 9628), and for the high fatigue model, training stopped after 103 epochs (iteration 12782). Models were implemented using the Machine Learning Toolbox in MATLAB R2023b.

After training the models, we computed the sensitivity matrix S for each fatigue state using the formula:

1 $$S=\frac{1}{N}{\sum }_{i=1}^{N}({\nabla }_{{x}_{i}}{y}_{i}){({\nabla }_{{x}_{i}}{y}_{i})}^{T},$$

where *N* is the number of samples, *y*_*i*_ is the model’s output for the *i*-th sample, *x*_*i*_ is the input vector for the i-th sample, and ${\nabla }_{{x}_{i}}{y}_{i}$ is the gradient of the model’s output with respect to the input vector *x*_*i*_, i.e.,

2 $${\nabla }_{{x}_{i}}{y}_{i}=\frac{\partial {y}_{i}}{\partial {x}_{i}}={\left[\frac{\partial {y}_{i}}{\partial {x}_{i,1}},\frac{\partial {y}_{i}}{\partial {x}_{i,2}},\ldots ,\frac{\partial {y}_{i}}{\partial {x}_{i,25}},\right]}^{{{\rm{T}}}}.$$

The sensitivity matrix captures how variations in input features affect the model’s output, providing a quantitative measure of feature importance and interactions. We then performed eigenvalue decomposition on S:

3 $$S={{\rm{V}}}\Lambda {{{\rm{V}}}}^{{{\rm{T}}}},$$

where V is a matrix, whose columns are the eigenvalues, and Λ is a diagonal matrix containing the eigenvalues. The eigenvalues were sorted in descending order. Larger eigenvalues correspond to directions in the input space where the output is more sensitive. The components of an eigenvector indicate the contribution of each feature to that sensitivity direction. We focused on the eigenvectors associated with the largest eigenvalues (explaining 90% of the variance) and computed the mean absolute values of their components to quantify the sensitivity contributions of different frequency bands. We compared the contribution values for the alpha and beta bands (Fig. 6D, left panel).

To investigate why tRNS effects were observed only under low fatigue, we statistically compared the sensitivity contributions of different frequency bands between low and high fatigue states using permutation tests. We combined all samples from both fatigue states and randomly permuted their labels 10000 times. In each permutation, we conducted the sensitivity analysis as described above to obtain the difference in contributions for each frequency band between low and high fatigue. We calculated *p*-values by determining the position of the observed difference within the distribution of permuted differences for each frequency band. False discovery rate (FDR) correction was applied for multiple comparisons.

To justify the hyperparameter combination, we optimized our neural network models by systematically varying key hyperparameters: the number of hidden units (32 and 64), batch sizes (32 and 64), and learning rates (0.01 and 0.001), resulting in eight different combinations. Early stopping with a patience of five epochs was employed to prevent overfitting. We selected these hyperparameters because they directly affect model capacity, training dynamics, and the ability to capture nonlinear relationships. We compared the validation loss across all combinations and selected the hyperparameter configuration that yielded the lowest validation loss for the final model due to its superior fit and generalization performance. This approach ensured that our model achieved optimal performance while maintaining robustness against overfitting. The comparison results can be found in Supplementary Table 17 and Fig. 4.

### Statistics and reproducibility

All statistical analyses were conducted using Bayesian linear mixed models and permutation-based sensitivity analysis. Bayesian models were implemented in R using the brms package and the Stan probabilistic programming language. Models included random intercepts for participants and, where feasible, random slopes to account for within-subject variability. Evidence was considered credible and interpreted as statistically significant under Bayesian criteria if the posterior probability (*Pr*) exceeded 97.5% and the 95% HPD interval excluded zero; otherwise, evidence was considered inconclusive^60,61^.

Sample size was determined a priori based on previous studies using similar paradigms and data modalities^12,29^. Of the 38 initially recruited participants, 29 (15 females; mean age: 22.7 ± 1.9 years) were retained after applying predefined exclusion criteria. Each participant completed both tRNS and sham conditions in a within-subject, counterbalanced design. Each condition comprised five experimental blocks, including a resting period (with or without stimulation) and a visual detection task, yielding EEG and fNIRS data across fatigue states. Trials were averaged within blocks to estimate VCT, and replicates were defined as within-subject, within-condition blocks or trials depending on the analysis.

Neural network models used for sensitivity analysis were trained separately on low- and high-fatigue sham trials to isolate perceptual relevance independent of tRNS effects. Permutation tests (10000 iterations) assessed the significance of observed differences in EEG frequency band contributions to VCT. All analyses and model comparisons were validated using cross-validation or held-out data, and hyperparameter selection was guided by minimum validation loss. Full model specifications and hyperparameter optimization details are provided in the Supplementary Information.

### Reporting summary

Further information on research design is available in the Nature Portfolio Reporting Summary linked to this article.

## Supplementary information

Supplementary Information

Reporting Summary

## Data Availability

The data and code to reproduce the results are available on the Open Science Framework at https://osf.io/4ynt3/.

## Code availability

The data and code to reproduce the results are available on the Open Science Framework at https://osf.io/4ynt3/.
