# Supplementary material for: Enhancing visual perception by modulating prestimulus alpha and beta power with tRNS
Source: Commun Biol. 2025 Aug 8;8:1182. doi: 10.1038/s42003-025-08600-z (PMC12334615; doi:10.1038/s42003-025-08600-z)
Supplement: Supplementary file 1 — Supplementary Information [file 42003_2025_8600_MOESM1_ESM.pdf]

# Supplemental Information

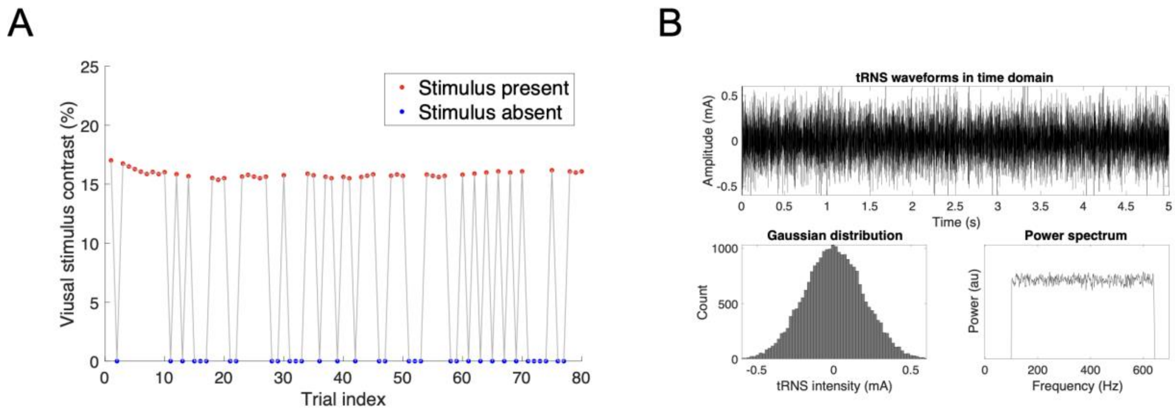


**Supplementary Fig. 1 A** Within each block, visual stimuli were presented in 60% of trials (denoted by red dots), while stimuli were absent in the remaining trials (denoted by blue dots). **B** The tRNS current waveform had intensities normally distributed, with 99% of values within the peak-to-peak amplitude of 1 mA. The power spectrum illustrates the high-frequency band (100–640 Hz) tRNS used in the study.

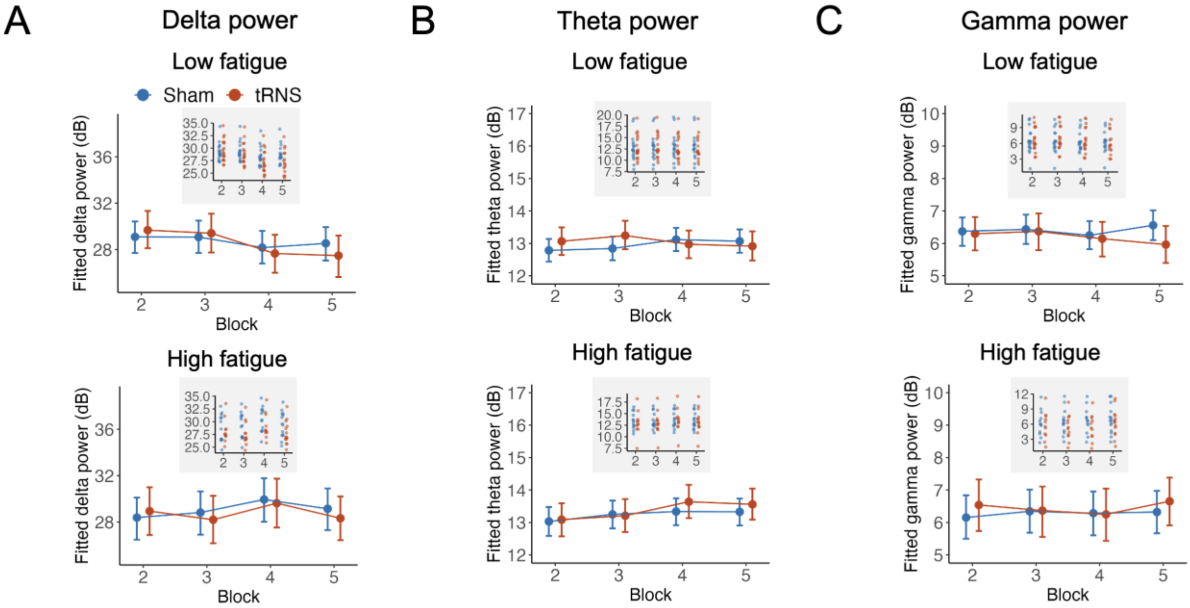


**Supplementary Fig. 2 Effects of tRNS on prestimulus delta, theta, and gamma power (see statistics in Table S14 – 16). A** tRNS did not affect prestimulus delta power under either low or high fatigue states. The inset plot with a grey background displays fitted individual data points. **B** tRNS did not affect prestimulus theta power

under either low or high fatigue states. **C** tRNS did not affect prestimulus gamma power either both low or high fatigue states.

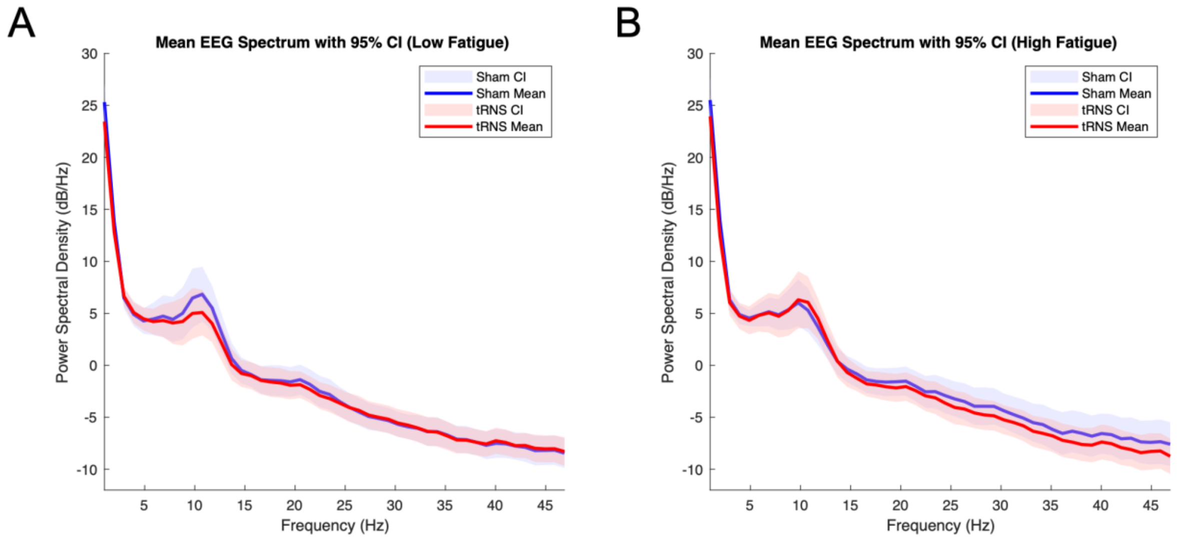


**Supplementary Fig. 3 Mean EEG power spectra with 95% confidence intervals (CI) for Block 5 under low and high fatigue conditions.** Spectral power density (in dB/Hz) is plotted across frequencies (1–45 Hz) for tRNS (red) and sham (blue) conditions, shown separately for low-fatigue (left) and high-fatigue (right) states. Shaded regions indicate 95% confidence intervals across participants. Spectra were computed using Welch's method (200-sample Hamming window, 50% overlap, 256-point FFT) and averaged across electrodes and trials for each participant. These plots provide descriptive spectral profiles to visualize overall frequency content differences between conditions. Note that these spectra reflect power per unit frequency (dB/Hz) and are therefore not directly comparable to the band power values (total power in a frequency range, typically expressed in dB) shown in Figures 4–5. The difference in scaling reflects this distinction in measurement type. A cluster-based permutation test revealed no significant differences between tRNS and sham conditions at either fatigue level (Maris and Oostenveld 2007). Furthermore, these raw spectral plots do not control for trial-level variability, baseline power, or block-wise trends. In contrast, the band-specific results presented in Fig. 4B–C are derived from Bayesian linear mixed-effects models that adjust for within-subject effects of stimulation condition, fatigue level, and block progression. These adjustments are essential for isolating condition-specific modulation in neuromodulation studies. While some apparent differences are visible in the raw spectra (e.g., elevated broadband power after 15 Hz under Sham in high-fatigue state), these patterns should not be interpreted as direct statistical evidence of the stimulation effects reported in the main text.

**Supplementary Table 1.** Effects of tRNS on fNIRS HbO amplitude (refer to Fig. 3A).

This table reported the contrast results of fNIRS HbO amplitude between sham and tRNS conditions obtained from Bayesian linear mixed model. It includes the estimated contrast coefficients (*Est*), the lower and upper ranges of the 95% highest probability density (Lower *HPD*: *LH*; Upper *HPD*: *UH*) for these estimates, and the posterior probability (*Pr*) indicating the presence of contrast difference. The hypotheses stating that contrast difference existed were formulated. The evidence was considered compelling to support the hypothesis if the

posterior probability exceeds 97.5% and 95% *HPD* does not include 0, as shown by bold line. For the associated linear mixed models, refer to the Methods section.

| Contrast | Block | *Est* | *LH* | *UH* | *Pr* |
|---|---|---|---|---|---|
| Sham - tRNS | 2 | –0.078 | –0.187 | 0.029 | 0.922 |
| Sham - tRNS | 3 | –0.107 | –0.213 | 0.006 | 0.970 |
| Sham - tRNS | 4 | –0.090 | –0.200 | 0.011 | 0.956 |
| **Sham - tRNS** | **5** | **–0.169** | **–0.270** | **–0.056** | **0.998** |

**Supplementary Table 2.** Effects of tRNS on alpha power (refer to Fig. 3B).

| Contrast | Block | *Est* | *LH* | *UH* | *Pr* |
|---|---|---|---|---|---|
| Sham - tRNS | 2 | –0.257 | –0.727 | 0.203 | 0.853 |
| Sham - tRNS | 3 | –0.144 | –0.629 | 0.307 | 0.730 |
| Sham - tRNS | 4 | –0.054 | –0.509 | 0.436 | 0.589 |
| Sham - tRNS | 5 | 0.093 | –0.396 | 0.566 | 0.350 |

**Supplementary Table 3.** Effects of tRNS on beta power (refer to Fig. 3C).

| Contrast | Block | *Est* | *LH* | *UH* | *Pr* |
|---|---|---|---|---|---|
| Sham - tRNS | 2 | –0.147 | –0.516 | 0.217 | 0.799 |
| Sham - tRNS | 3 | –0.044 | –0.386 | 0.333 | 0.594 |
| Sham - tRNS | 4 | 0.078 | –0.291 | 0.431 | 0.670 |
| Sham - tRNS | 5 | 0.142 | –0.203 | 0.523 | 0.780 |

**Supplementary Table 4.** Effects of tRNS on VCT (refer to Fig. 3D).

| Contrast | Block | *Est* | *LH* | *UH* | *Pr* |
|---|---|---|---|---|---|
| Sham - tRNS | 2 | 0.042 | –0.229 | 0.324 | 0.623 |
| Sham - tRNS | 3 | 0.181 | –0.079 | 0.459 | 0.913 |
| Sham - tRNS | 4 | 0.207 | –0.069 | 0.472 | 0.944 |

| Contrast | Block | *Est* | *LH* | *UH* | *Pr* |
|---|---|---|---|---|---|
| Sham - tRNS | 5 | 0.184 | –0.085 | 0.448 | 0.922 |

**Supplementary Table 5.** Effects of tRNS on fatigue ratings (refer to Fig. 3E).

| Contrast | Block | *Est* | *LH* | *UH* | *Pr* |
|---|---|---|---|---|---|
| Sham - tRNS | 2 | –0.034 | –0.234 | 0.141 | 0.639 |
| Sham - tRNS | 3 | –0.008 | –0.214 | 0.224 | 0.527 |
| Sham - tRNS | 4 | 0.186 | –0.209 | 0.598 | 0.824 |
| Sham - tRNS | 5 | 0.088 | –0.319 | 0.518 | 0.672 |

**Supplementary Table 6.** Effects of tRNS on fNIRS HbO amplitude under different fatigue states (refer to Fig. 4A).

This table reported the contrast results of fNIRS HbO amplitude under low and high fatigue states between sham and tRNS conditions obtained from Bayesian linear mixed model.

| | | HbO: Low fatigue | | | | HbO: High fatigue | | | |
|---|---|---|---|---|---|---|---|---|---|
| Contrast | Block | *Est* | *LH* | *UH* | *Pr* | *Est* | *LH* | *UH* | *Pr* |
| Sham - tRNS | 2 | –0.096 | –0.230 | 0.028 | 0.930 | –0.035 | –0.197 | 0.113 | 0.673 |
| Sham - tRNS | 3 | –0.133 | –0.276 | –0.005 | 0.974 | –0.071 | –0.210 | 0.072 | 0.839 |
| Sham - tRNS | 4 | –0.128 | –0.263 | 0.001 | 0.969 | –0.058 | 0.206 | 0.088 | 0.781 |
| **Sham - tRNS** | **5** | **–0.276** | **–0.405** | **–0.137** | **1** | –0.039 | –0.183 | 0.109 | 0.706 |

**Supplementary Table 7.** Effects of tRNS on **alpha** power under different fatigue states (refer to Fig. 4B).

This table reported the contrast results of alpha power under low and high fatigue states between sham and tRNS conditions obtained from Bayesian linear mixed model.

| | | Alpha power: Low fatigue | | | | Alpha power: High fatigue | | | |
|---|---|---|---|---|---|---|---|---|---|
| Contrast | Block | *Est* | *LH* | *UH* | *Pr* | *Est* | *LH* | *UH* | *Pr* |
| Sham - tRNS | 2 | –0.354 | –0.897 | 0.195 | 0.900 | –0.124 | –0.744 | 0.542 | 0.650 |
| Sham - tRNS | 3 | –0.129 | –0.645 | 0.423 | 0.677 | –0.248 | –0.899 | 0.368 | 0.786 |

| | | | | | | | | | |
|---|---|---|---|---|---|---|---|---|---|
| Sham - tRNS | 4 | 0.330 | −0.224 | 0.873 | 0.882 | −0.633 | −0.253 | 0.032 | 0.972 |
| **Sham - tRNS** | **5** | **0.582** | **0.034** | **1.162** | **0.977** | −0.453 | −1.013 | 0.156 | 0.930 |

**Supplementary Table 8.** Effects of tRNS on **beta** power under different fatigue states (refer to Fig. 4C). This table reported the contrast results of beta power under low and high fatigue states between sham and tRNS conditions obtained from Bayesian linear mixed model.

| | | Beta power: Low fatigue | | | | Beta power: High fatigue | | | |
|---|---|---|---|---|---|---|---|---|---|
| Contrast | Block | *Est* | *LH* | *UH* | *Pr* | *Est* | *LH* | *UH* | *Pr* |
| Sham - tRNS | 2 | −0.107 | −0.510 | 0.307 | 0.698 | −0.229 | −0.809 | 0.326 | 0.819 |
| Sham - tRNS | 3 | −0.013 | −0.433 | 0.441 | 0.477 | −0.080 | −0.628 | 0.475 | 0.621 |
| Sham - tRNS | 4 | 0.297 | −0.112 | 0.725 | 0.919 | −0.245 | −0.798 | 0.333 | 0.800 |
| **Sham - tRNS** | **5** | **0.482** | **0.047** | **0.927** | **0.983** | −0.209 | −0.755 | 0.330 | 0.782 |

**Supplementary Table 9.** Effects of tRNS on **VCT** under different fatigue states (refer to Fig. 4D). This table reported the contrast results of VCT under low and high fatigue states between sham and tRNS conditions obtained from Bayesian linear mixed model.

| | | VCT: Low fatigue | | | | VCT: High fatigue | | | |
|---|---|---|---|---|---|---|---|---|---|
| Contrast | Block | *Est* | *LH* | *UH* | *Pr* | *Est* | *LH* | *UH* | *Pr* |
| Sham - tRNS | 2 | 0.015 | −0.273 | 0.309 | 0.546 | 0.053 | −0.308 | 0.402 | 0.617 |
| Sham - tRNS | 3 | 0.163 | −0.116 | 0.462 | 0.862 | 0.229 | −0.119 | 0.581 | 0.900 |
| Sham - tRNS | 4 | 0.245 | −0.049 | 0.551 | 0.952 | 0.206 | −0.154 | 0.556 | 0.871 |
| **Sham - tRNS** | **5** | **0.307** | **0.001** | **0.603** | **0.978** | 0.092 | −0.258 | 0.414 | 0.702 |

**Supplementary Table 10.** Effects of fatigue state on fNIRS HbO amplitude under different stimulation conditions (refer to Fig. 5A). This table reported the contrast results of fNIRS HbO amplitude under sham and tRNS conditions between low and high fatigue states obtained from Bayesian linear mixed model.

| | | fNIRS HbO amplitude: Sham | | | | fNIRS HbO amplitude: tRNS | | | |
|---|---|---|---|---|---|---|---|---|---|
| Contrast | Block | *Est* | *LH* | *UH* | *Pr* | *Est* | *LH* | *UH* | *Pr* |
| Low - High | 2 | −0.071 | −0.199 | 0.054 | 0.871 | −0.013 | −0.157 | 0.132 | 0.572 |

| | | | | | | | | | |
|---|---|---|---|---|---|---|---|---|---|
| Low - High | 3 | –0.068 | –0.195 | 0.063 | 0.841 | –0.004 | –0.148 | 0.145 | 0.519 |
| Low - High | 4 | –0.066 | –0.190 | 0.067 | 0.844 | 0.007 | –0.128 | 0.165 | 0.538 |
| Low - High | 5 | –0.110 | –0.229 | 0.021 | 0.955 | 0.128 | –0.019 | 0.263 | 0.961 |

**Supplementary Table 11.** Effects of fatigue state on **alpha** power under different stimulation conditions (refer to Fig. 5B).

This table reported the contrast results of alpha power under sham and tRNS conditions between low and high fatigue states obtained from Bayesian linear mixed model.

| | | Alpha power: Sham | | | | Alpha power: tRNS | | | |
|---|---|---|---|---|---|---|---|---|---|
| Contrast | Block | *Est* | *LH* | *UH* | *Pr* | *Est* | *LH* | *UH* | *Pr* |
| Low - High | 2 | –0.226 | –0.759 | 0.308 | 0.806 | –0.004 | –0.647 | 0.542 | 0.503 |
| Low - High | 3 | –0.240 | –0.808 | 0.295 | 0.812 | –0.365 | –0.989 | 0.203 | 0.892 |
| Low - High | **4** | 0.134 | –0.429 | 0.640 | 0.696 | **–0.819** | **–1.445** | **–0.261** | **0.996** |
| Low - High | **5** | 0.171 | –0.351 | 0.703 | 0.739 | **–0.850** | **–1.438** | **–0.282** | **0.997** |

**Supplementary Table 12.** Effects of fatigue state on **beta** power under different stimulation conditions (refer to Fig. 5C).

This table reported the contrast results of beta power under sham and tRNS conditions between low and high fatigue states obtained from Bayesian linear mixed model.

| | | Beta power: Sham | | | | Beta power: tRNS | | | |
|---|---|---|---|---|---|---|---|---|---|
| Contrast | Block | *Est* | *LH* | *UH* | *Pr* | *Est* | *LH* | *UH* | *Pr* |
| Low - High | 2 | –0.046 | –0.482 | 0.399 | 0.576 | –0.174 | –0.647 | 0.313 | 0.760 |
| Low - High | 3 | –0.082 | –0.516 | 0.359 | 0.650 | –0.138 | –0.613 | 0.340 | 0.718 |
| Low - High | **4** | 0.043 | –0.409 | 0.483 | 0.418 | **–0.491** | **–0.981** | **–0.005** | **0.975** |
| Low - High | **5** | 0.119 | –0.330 | 0.535 | 0.709 | **–0.570** | **–1.075** | **–0.124** | **0.989** |

**Supplementary Table 13.** Effects of fatigue state on **VCT** under different stimulation conditions (refer to Fig. 5D).

This table reported the contrast results of VCT under sham and tRNS conditions between low and high fatigue states obtained from Bayesian linear mixed model.

| | VCT: Sham | VCT: tRNS |
|---|---|---|

| Contrast | Block | *Est* | *LH* | *UH* | *Pr* | *Est* | *LH* | *UH* | *Pr* |
|---|---|---|---|---|---|---|---|---|---|
| Low - High | 2 | −0.063 | −0.374 | 0.223 | 0.662 | −0.019 | −0.326 | 0.309 | 0.546 |
| Low - High | 3 | −0.054 | −0.371 | 0.245 | 0.645 | 0.027 | −0.299 | 0.323 | 0.567 |
| Low - High | 4 | −0.061 | −0.387 | 0.228 | 0.661 | −0.060 | −0.386 | 0.248 | 0.653 |
| Low - High | 5 | −0.013 | −0.319 | 0.274 | 0.533 | −0.212 | −0.506 | 0.111 | 0.915 |

**Supplementary Table 14.** Effects of tRNS on **delta power** under different fatigue states (refer to Fig. S2_A). This table reported the contrast results of delta power under low and high fatigue states between sham and tRNS conditions obtained from Bayesian linear mixed model.

| | | Delta power: Low fatigue | | | | Delta power: High fatigue | | | |
|---|---|---|---|---|---|---|---|---|---|
| Contrast | Block | *Est* | *LH* | *UH* | *Pr* | *Est* | *LH* | *UH* | *Pr* |
| Sham - tRNS | 2 | −0.542 | −2.396 | 1.616 | 0.697 | −0.574 | −3.256 | 2.082 | 0.665 |
| Sham - tRNS | 3 | −0.322 | −2.312 | 1.742 | 0.621 | 0.608 | −2.189 | 3.153 | 0.675 |
| Sham - tRNS | 4 | 0.554 | −1.529 | 2.648 | 0.700 | 0.286 | −2.492 | 2.899 | 0.584 |
| Sham - tRNS | 5 | 1.047 | −1.025 | 3.206 | 0.838 | 0.825 | −1.760 | 3.232 | 0.746 |

**Supplementary Table 15.** Effects of tRNS on **theta power** under different fatigue states (refer to Fig. S2_B). This table reported the contrast results of delta power under low and high fatigue states between sham and tRNS conditions obtained from Bayesian linear mixed model.

| | | Theta power: Low fatigue | | | | Theta power: High fatigue | | | |
|---|---|---|---|---|---|---|---|---|---|
| Contrast | Block | *Est* | *LH* | *UH* | *Pr* | *Est* | *LH* | *UH* | *Pr* |
| Sham - tRNS | 2 | −0.291 | −0.829 | 0.234 | 0.858 | −0.058 | −0.735 | 0.593 | 0.568 |
| Sham - tRNS | 3 | −0.405 | −0.982 | 0.098 | 0.932 | 0.042 | −0.607 | 0.683 | 0.548 |
| Sham - tRNS | 4 | 0.136 | −0.366 | 0.685 | 0.701 | −0.326 | −0.970 | 0.350 | 0.839 |
| Sham - tRNS | 5 | 0.142 | −0.383 | 0.708 | 0.691 | −0.242 | −0.854 | 0.355 | 0.777 |

**Supplementary Table 16.** Effects of tRNS on **gamma power** under different fatigue states (refer to Fig. S2_C).

This table reported the contrast results of gamma power under low and high fatigue states between sham and tRNS conditions obtained from Bayesian linear mixed model.

| | | Gamma power: Low fatigue | | | | Gamma power: High fatigue | | | |
|---|---|---|---|---|---|---|---|---|---|
| Contrast | Block | *Est* | *LH* | *UH* | *Pr* | *Est* | *LH* | *UH* | *Pr* |
| Sham - tRNS | 2 | 0.057 | –0.549 | 0.678 | 0.572 | –0.363 | –1.285 | 0.582 | 0.781 |
| Sham - tRNS | 3 | 0.059 | –0.547 | 0.718 | 0.572 | –0.015 | –0.889 | 0.924 | 0.512 |
| Sham - tRNS | 4 | 0.120 | –0.490 | 0.766 | 0.644 | 0.050 | –0.914 | 0.982 | 0.541 |
| Sham - tRNS | 5 | 0.586 | –0.076 | 1.271 | 0.961 | –0.325 | –1.261 | 0.498 | 0.772 |

## Hyperparameter Selection in Sensitivity Analysis

To ensure the robustness and reliability of our neural network models, we conducted a systematic exploration of key hyperparameters. Specifically, we varied the number of hidden units (32 and 64), batch sizes (32 and 64), and learning rates (0.01 and 0.001), resulting in a total of eight combinations. The early stopping patience was consistently set to 5 epochs across all experiments.

The selection of 32 and 64 hidden units was based on balancing model complexity with the risk of overfitting, considering our input size of 25 features. Choosing hidden layer sizes that are not excessively larger than the input size helps prevent over-parameterization and ensures better generalization to unseen data[1]. This aligns with established guidelines suggesting that the number of hidden units should be proportional to the input size to maintain computational efficiency and training stability[2]. Larger hidden layer sizes, such as 128 units, were avoided to reduce the risk of overfitting given our dataset size and to maintain computational efficiency[3].

Batch sizes of 32 and 64 were chosen to balance computational efficiency and convergence speed. Smaller batch sizes can lead to noisy gradient estimates, while larger batch sizes may require more memory and could potentially slow down convergence[4]. By selecting these batch sizes, we aimed to achieve efficient training while maintaining the quality of gradient updates.

We experimented with learning rates of 0.01 and 0.001 to explore the impact of different optimization speeds on model performance. A higher learning rate like 0.01 allows for faster convergence but may risk overshooting minima, while a lower learning rate like 0.001 provides

more stable convergence at the expense of longer training times[5]. Exploring both values enabled us to assess the sensitivity of our models to this critical hyperparameter.

The early stopping patience was set to 5 epochs based on recommendations from the literature and standard practices observed in machine learning frameworks. This choice balances the need for sufficient training time to allow the model to improve with the goal of preventing overfitting and ensuring efficient use of computational resources[6,7]. Setting the patience to 5 prevents the early stopping mechanism from being too sensitive to minor fluctuations in validation loss, allowing the training process to focus on longer-term trends in model improvement.

We evaluated each hyperparameter combination by comparing the validation loss obtained during training (Supplementary Table 13). The combination that resulted in the lowest validation loss was selected for the final model due to its better fit and generalization performance. This approach allowed us to identify the most effective hyperparameter configuration for our data, ensuring that the model achieves optimal performance while avoiding overfitting. We found that when batch size equals 32 or 64 and other hyperparameters were fixed, the validation losses were quite comparable (Supplementary Table 13). Therefore, we also tried the batch size of 64 and performed the same procedures for sensitivity analysis (Supplementary Fig. 2). The results were consistent with Fig. 6.

By systematically varying these hyperparameters and evaluating model performance based on validation loss, we ensured that our findings are robust and not artifacts of specific hyperparameter choices.

**Supplementary Table 17.** Comparison of validation loss across different hyperparameter combinations. This table reported the validation losses by using different hyperparameter combinations in neural network modelling of the sensitivity analysis. The bold rows yielded comparable results.

| Hidden size | Batch size | Learning rate | Validation loss |
|---|---|---|---|
| 32 | 32 | 0.01 | Low: 0.155; high: 0.167 |
| 32 | 64 | 0.01 | Low: 0.153; high: 0.177 |
| 32 | 32 | 0.001 | Low: 0.139; high: 0.162 |
| 32 | 64 | 0.001 | Low: 0.140; high: 0.159 |

| | | | |
|---|---|---|---|
| 64 | 32 | 0.01 | Low: 0.152; high: 0.167 |
| 64 | 64 | 0.01 | Low: 0.135; high: 0.164 |
| **64** | **32** | **0.001** | **Low: 0.130; high: 0.142** |
| **64** | **64** | **0.001** | **Low: 0.125; high: 0.150** |

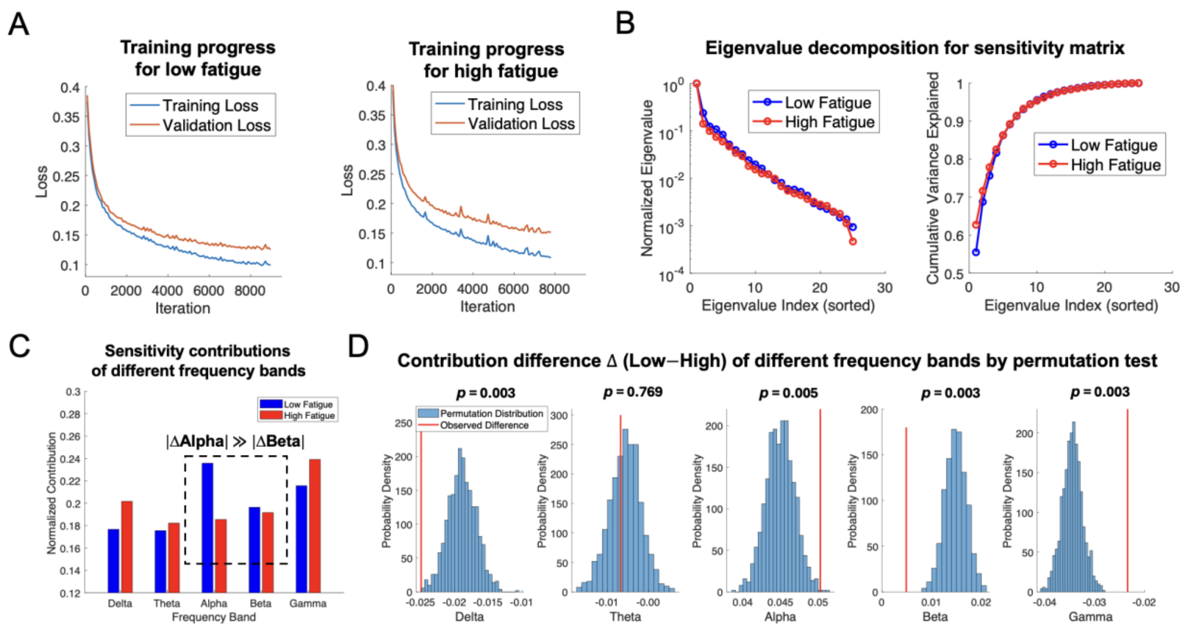


**Supplementary Fig. 4** Sensitivity analysis for another hyperparameter combination candidate: hidden size = 64, batch size = 64, learning rate = 0.001, patience = 5. **A** To prevent overfitting, samples under each fatigue state were divided into training and validation subsets. Early stopping was employed during model training. **B** Eigenvalue decomposition of the sensitivity matrix was performed. Eigenvalues explaining 90% of the variance were selected to calculate the sensitivity contributions of different frequency bands to VCT. **C** The alpha band showed a larger sensitivity shift than the beta band from low to high fatigue states. **D** Permutation distributions of Δ = Low–High for each band. Red line = observed Δ. Two-tailed *p*-values (FDR-corrected) appear above each panel. Significant differences emerged for the delta ($p$ = 0.003), alpha ($p$ = 0.005), beta ($p$ = 0.003), and gamma ($p$ = 0.003) bands, while theta showed no reliable change ($p$ = 0.769). Notably, the alpha band exhibited the largest positive shift, indicating substantially greater perceptual relevance under low-fatigue. In contrast, although the beta band difference was statistically significant, its magnitude was much smaller than that of alpha, suggesting that while beta may contribute modestly to state-dependent perception, alpha plays the dominant role. Delta and gamma showed increased contributions under high-fatigue, suggesting a compensatory redistribution of perceptual weighting. The consistency of

these findings across model configurations underscores the robustness of alpha-band sensitivity as the primary state-dependent driver of perceptual performance.

## Reference


1. Goodfellow, I., Bengio, Y., & Courville, A. (2016). *Deep Learning*. MIT Press.
2. Heaton, J. (2017). *Artificial Intelligence for Humans, Volume 3: Deep Learning and Neural Networks*. Heaton Research.
3. Hawkins, D. M. (2004). "The Problem of Overfitting." *Journal of Chemical Information and Computer Sciences*, 44(1), 1–12.
4. Wilson, A. C., et al. (2017). "The Marginal Value of Adaptive Gradient Methods in Machine Learning." *Advances in Neural Information Processing Systems*, 30.
5. Bengio, Y. (2012). "Practical Recommendations for Gradient-Based Training of Deep Architectures." In *Neural Networks: Tricks of the Trade* (pp. 437–478). Springer.
6. Prechelt, L. (2002). "Early Stopping — But When?" In *Neural Networks: Tricks of the Trade* (pp. 55–69). Springer.
7. Brownlee, J. (2018). "How to Stop Training Deep Neural Networks At the Right Time Using Early Stopping." *Machine Learning Mastery*.
