# Supplementary material for: Enhancing visual perception by modulating prestimulus alpha and beta power with tRNS
Source: Commun Biol. 2025 Aug 8;8:1182. doi: 10.1038/s42003-025-08600-z (PMC12334615; doi:10.1038/s42003-025-08600-z)
Supplement: Supplementary file 2 — Reporting Summary [file 42003_2025_8600_MOESM2_ESM.pdf]

nature portfolio

Corresponding author(s): DAPR COMMSBIO-25-0364B

Last updated by author(s): Jul 24, 2025

Reporting Summary

Nature Portfolio wishes to improve the reproducibility of the work that we publish. This form provides structure for consistency and transparency in reporting. For further information on Nature Portfolio policies, see our Editorial Policies and the Editorial Policy Checklist.

Statistics

For all statistical analyses, confirm that the following items are present in the figure legend, table legend, main text, or Methods section.

n/a	Confirmed	
☐	☒	The exact sample size (n) for each experimental group/condition, given as a discrete number and unit of measurement
☐	☒	A statement on whether measurements were taken from distinct samples or whether the same sample was measured repeatedly
☐	☒	The statistical test(s) used AND whether they are one- or two-sided<br>*Only common tests should be described solely by name; describe more complex techniques in the Methods section.*
☐	☒	A description of all covariates tested
☐	☒	A description of any assumptions or corrections, such as tests of normality and adjustment for multiple comparisons
☐	☒	A full description of the statistical parameters including central tendency (e.g. means) or other basic estimates (e.g. regression coefficient) AND variation (e.g. standard deviation) or associated estimates of uncertainty (e.g. confidence intervals)
☐	☒	For null hypothesis testing, the test statistic (e.g. F, t, r) with confidence intervals, effect sizes, degrees of freedom and P value noted<br>*Give P values as exact values whenever suitable.*
☐	☒	For Bayesian analysis, information on the choice of priors and Markov chain Monte Carlo settings
☐	☒	For hierarchical and complex designs, identification of the appropriate level for tests and full reporting of outcomes
☐	☒	Estimates of effect sizes (e.g. Cohen's d, Pearson's r), indicating how they were calculated

Our web collection on statistics for biologists contains articles on many of the points above.

Software and code

Policy information about availability of computer code

Data collection	Commercial and open-source software were used for data collection. Specifically:<br><br>MATLAB R2023b (MathWorks, Natick, MA): Used for implementing the visual detection task with Psychophysics Toolbox 3.<br><br>BrainVision Recorder (Brain Products GmbH): Used for EEG signal acquisition.<br><br>NIRStar (NIRx Medical Technologies, LLC): Used for fNIRS signal acquisition from the NIRSport2 system.<br><br>Soterix Medical software: Used to control the transcranial random noise stimulation (tRNS) device.
Data analysis	A combination of commercial, open-source, and custom code was used:<br><br>MATLAB R2023b: Used for EEG preprocessing, power spectral analysis, neural network modeling, and sensitivity analysis.<br><br>EEGLAB Toolbox (Delorme & Makeig, 2004): Used for EEG preprocessing including filtering, epoching, and artifact rejection.<br><br>Bandpower and Pwelch functions: MATLAB built-in functions used to compute EEG band power.<br><br>R (version 4.4.2) with the brms package (Bürkner, 2017): Used for Bayesian linear mixed-effects modeling via Stan.<br><br>Custom MATLAB scripts were written for the sensitivity matrix computation, eigen decomposition, and permutation testing.

The full analysis pipeline and code are available at: https://osf.io/4ynt3/.

For manuscripts utilizing custom algorithms or software that are central to the research but not yet described in published literature, software must be made available to editors and reviewers. We strongly encourage code deposition in a community repository (e.g. GitHub). See the Nature Portfolio guidelines for submitting code & software for further information.

Data

Policy information about availability of data

All manuscripts must include a data availability statement. This statement should provide the following information, where applicable:

- Accession codes, unique identifiers, or web links for publicly available datasets
- A description of any restrictions on data availability
- For clinical datasets or third party data, please ensure that the statement adheres to our policy

The data and code to reproduce the results are available on the Open Science Framework at https://osf.io/4ynt3/.

Research involving human participants, their data, or biological material

Policy information about studies with human participants or human data. See also policy information about sex, gender (identity/presentation), and sexual orientation and race, ethnicity and racism.

Reporting on sex and gender	Sex was recorded as a binary variable (male/female) based on self-report. Of the 29 participants, 15 were female and 14 were male. Gender was not separately collected or analyzed. The study was not designed or powered to assess sex- or gender-based differences, and no sex-specific analyses were performed.
Reporting on race, ethnicity, or other socially relevant groupings	No data on race, ethnicity, or other socially relevant groupings were collected or analyzed. These variables were not considered relevant to the hypotheses or objectives of the study and thus were not included in recruitment or analysis. No such group-based confounders were examined.
Population characteristics	Participants were right-handed, healthy university students (mean age: 22.7 ± 1.9 years) with normal or corrected-to-normal vision. They had no history of neurological, psychiatric, or cognitive disorders, and were not on medications affecting the central nervous system. See above for sex-related demographics.
Recruitment	Participants were recruited via university mailing lists and campus advertisements. All participants provided written informed consent. Exclusion criteria included left-handedness, history of seizures or head injuries, use of psychotropic medication, implanted electronic devices, metal implants in the head, psychiatric disorders, tinnitus, or prior neuromodulation participation within the past three months. Self-selection bias is a possibility, given the voluntary nature of recruitment, but was minimized through strict screening procedures.
Ethics oversight	This study was approved by the ethics committee at Shenzhen University. All procedures complied with the Declaration of Helsinki.

Note that full information on the approval of the study protocol must also be provided in the manuscript.

Field-specific reporting

Please select the one below that is the best fit for your research. If you are not sure, read the appropriate sections before making your selection.

☒ Life sciences ☐ Behavioural & social sciences ☐ Ecological, evolutionary & environmental sciences

For a reference copy of the document with all sections, see nature.com/documents/nr-reporting-summary-flat.pdf

Life sciences study design

All studies must disclose on these points even when the disclosure is negative.

Sample size	The sample size was determined based on precedent from similar published studies using tRNS with EEG/fNIRS in perceptual paradigms (e.g., Wei et al., 2022, 2024). No formal power analysis was performed. A total of 38 participants were initially recruited, of which 29 completed the study and were included in the final analysis. This sample size was considered sufficient to detect within-subject effects using Bayesian linear mixed-effects models.
Data exclusions	Nine participants were excluded prior to analysis due to: misunderstanding of instructions, excessive EEG/fNIRS artifacts, or discomfort during stimulation. These criteria were pre-established in the study protocol and approved by the ethics committee. No additional data exclusions were made post hoc.
Replication	All experiments were conducted using a within-subject, counterbalanced design with both sham and tRNS conditions. Each participant underwent both conditions across two separate sessions. Findings were internally replicated across five experimental blocks and two fatigue states. No findings reported in the manuscript failed to replicate within the design.
Randomization	Participants were randomly assigned to receive either tRNS or sham stimulation first, with the order counterbalanced across subjects. This

Randomization	within-subject design minimized between-subject variability. Fatigue state was classified independently for each block based on self-reported ratings, and not influenced by stimulation condition.
Blinding	The experiment was conducted in a single-blind fashion: participants were unaware of whether they received tRNS or sham stimulation. Due to technical limitations, investigators were aware of the condition but did not interact with participants during data collection. EEG was recorded after tRNS ended to eliminate artifact contamination, and data were preprocessed blindly using automated pipelines.

Reporting for specific materials, systems and methods

We require information from authors about some types of materials, experimental systems and methods used in many studies. Here, indicate whether each material, system or method listed is relevant to your study. If you are not sure if a list item applies to your research, read the appropriate section before selecting a response.

Materials & experimental systems

n/a	Involved in the study
☒	☐ Antibodies
☒	☐ Eukaryotic cell lines
☒	☐ Palaeontology and archaeology
☒	☐ Animals and other organisms
☒	☐ Clinical data
☒	☐ Dual use research of concern
☒	☐ Plants

Methods

n/a	Involved in the study
☒	☐ ChIP-seq
☒	☐ Flow cytometry
☒	☐ MRI-based neuroimaging

Plants

Seed stocks	n/a
Novel plant genotypes	n/a
Authentication	n/a
